# T Cell Metabolism in Infection

**DOI:** 10.3389/fimmu.2022.840610

**Published:** 2022-03-14

**Authors:** Jonas Aakre Wik, Bjørn Steen Skålhegg

**Affiliations:** Division for Molecular Nutrition, Institute of Basic Medical Sciences, University of Oslo, Oslo, Norway

**Keywords:** T cells, metabolism, immunometabolism, infection, HIV, COVID-19, tuberculosis

## Abstract

T lymphocytes (T cells) are divided into two functionally different subgroups the CD4+ T helper cells (Th) and the CD8+ cytotoxic T lymphocytes (CTL). Adequate CD4 and CD8 T cell activation to proliferation, clonal expansion and effector function is crucial for efficient clearance of infection by pathogens. Failure to do so may lead to T cell exhaustion. Upon activation by antigen presenting cells, T cells undergo metabolic reprograming that support effector functions. In this review we will discuss how metabolic reprograming dictates functionality during viral infections using severe acute respiratory syndrome coronavirus 2 (SARS-CoV-2) and human immunodeficiency virus (HIV) as examples. Moreover, we will briefly discuss T cell metabolic programs during bacterial infections exemplified by *Mycobacterium tuberculosis* (MT) infection.

## T Lymphocytes and Their Subsets Are Pivotal Cells of the Immune System

All multicellular organisms are in an “arms race” against infectious pathogens, which include pathogenic bacteria, viruses, fungi and parasites (1). The primary defenses against infectious pathogens are the physical and chemical barriers of the skin and mucosa, which separates the external and internal environments (1). Breach of these barriers allow pathogens to enter the body, requiring activation of the immune system to clear the infection (2). The immune system is a host defense system comprising many biological structures and processes within an organism that defends against foreign infection as well as damaged and transformed cells. The ability of the immune system to act optimally depends on its capacity to distinguish foreign and self and to react to non-self. In higher organisms, the immune system is classified into the innate and adaptive immune system (3). The innate immune system is rapidly engaged in an unspecific manner to foreign pathogens or damaged self through recognition of pathogen-associated molecular patterns (PAMP) or damage-associated molecular patterns (DAMP) (4, 5). In contrast to the innate immune system, the adaptive immune system is activated over a longer time-period and is associated with controlled activation of T- and B lymphocytes (T- and B cells), with immense specificity towards its targets, and immunological memory (5). B cells are activated and differentiate into plasma cells that produce immunoglobulins (Ig), commonly referred to as antibodies, following interaction between soluble antigens binding to the B cell receptor (BCR). T cells on the other hand are activated through cell-to-cell interactions when the T cell antigen receptor (TCR) complex encounters peptide antigens presented to them by antigen presenting cells (APC). APCs present antigens through major histocompatibility complex I or II (MHCI and MHCII), which interact with the two major subsets of T cells, CD8 positive (CD8+) and CD4 positive (CD4+) T cells, respectively. CD8+ T cells are also called cytotoxic T lymphocytes (CTLs) while CD4+ T cells are designated as T helper cells (Th) (2). The CTLs target virus-infected cells and induce cell death by three mechanisms. The secretion of proinflammatory cytokines, interaction between the Fas ligand and Fas receptor, and the secretion of cytolytic granules containing perforin, which creates pores in the target cell allowing the entry of proteases, including Granzyme B, that induce apoptosis (6, 7). CD4+ T cells are indirectly involved in clearing infection by modulating the activity of other immune cells, including macrophages (Mø), neutrophils, B cells and CTLs (2). CD4+ T cells can be grouped into several subsets and include T helper (Th) 1 (Th1), Th2, Th9, Th17, Th22 as well as follicular helper T (Tfh) cells and regulatory T cells (Tregs). The CD4+ T cell subsets are defined by the distinct expression of surface molecules and endogenous production of cytokines, which are driven by the presence of extracellular cytokines and activation of key transcription factors (8). Tfh cells are primarily involved in promoting survival, proliferation and class-switching of germinal center B cells and support germinal center development. Th1, Th2, Th9, Th17, and Th22 subsets, on the other hand, are involved in host defense against specific microbial pathogens. The Th1 and Th2 subsets were the first Th cell subsets to be identified. Whereas Th1 cells kill intracellular bacteria through activating Mø and CTLs, Th2 cells are drivers of immune reactions directed against extracellular parasites (8, 9). Th17 cells, which are characterized by the transcription factor retinoic acid receptor (RAR)-related orphan receptor γ (RORγ) and production of the cytokines IL-17A, IL-17F, IL-21 and IL-22, are involved in protection against pathogens on mucosal surfaces (8, 10–12). Th22 cells also secrete IL-22, but in contrast to Th17 cells, do not secrete IL-17 nor express RORγ (13). As Th22 cells are mainly found in the skin where IL-22 induces expression of antimicrobial peptides in keratinocytes and epithelial cells they are likely involved in maintaining homeostasis in skin (13, 14). Th9 cells, which are characterized by the secretion of IL-9, are involved in immune responses towards extracellular parasites, allergic inflammation and anti-tumor immune response. Interestingly, the anti-tumor functions of Th9 cells were found to be superior as compared to Th1 and other Th subsets and involves activation of the innate and adaptive immune system, including, generation of a profound CTL response against neo antigens (15, 16). Recently, it has also become evident that CD4+ subset of T cells can be cytotoxic themselves. These cytotoxic CD4+ T cells can induce cell death through interacting with peptides presented on MHC II, similar to CD8+ CTLs (17). While the CD4+ subsets are crucial in clearing infection, dysregulation may also result in pathological conditions including autoimmune diseases, allergy and asthma (14, 15, 18, 19). While Th1 and Th17 cells are implicated in autoimmunity and Th2 cells are involved in allergic immune responses, Th22 cells appear to be involved in both autoimmunity and allergy (14, 20–22). Th9 cells have recently been implicated in tumor immunity and promoting tolerance to transplanted organs (15, 23, 24). The activities of T cell subsets are balanced in part by unique CD4+ Treg T cell subpopulation. Tregs are vital to immune homeostasis and self-tolerance, dampening inflammation, and preventing the development of autoimmune disease, but may also be involved in promoting cancer progression (25). The balance between pro-inflammatory and anti-inflammatory signals is critically important.

It is widely accepted that the fundamental processes in T cell biology, such as T cell activation, differentiation and effector functions are closely linked to changes in the cellular metabolic programs. Key metabolic pathways such as glycolysis, fatty acid synthesis and mitochondrial metabolism play a crucial role in T cell immunometabolism (26).

## T Cell Metabolism: From Quiescence to Effector Function

T cell metabolism is crucial for maintaining homeostasis in naïve and memory cells, while also priming cells for rapid activation. Additionally, T cell metabolism drives and mirrors the activation and differentiation states of T cells. The focus of this section will be on how activation of T cells and metabolism are related as well as how metabolic reprograming drives the phenotype of activated T cells.

Naïve T cells enter the circulation from the thymus and are actively maintained in a reversible form of cell cycle arrest by a combination of self-peptide–MHC engagement of the TCR/CD3 and by interleukin (IL)-7 stimulation (27). Activation of T cells leads to exit of the quiescent state, inducing cell growth, clonal expansion and differentiation. This process is initiated and regulated by three key factors, perturbation of cell surface receptors, nutrient availability and oxygen levels (28).

The magnitude of acute T cell activation depends on activation of the TCR and co-stimulation of a group of T cell surface co-receptors. These co-receptors include the CD3, CD4, CD8 CD28/CTLA4 and more (29). As mentioned, the CD4 and CD8 molecules directly interact with the MHC II and MHC I molecules, respectively, and influence the early mode of T cell activation. Interaction between the MHC-peptide complex and CD4/CD8 co-receptors is recognized by the TCR, leading to activation of a signaling complex composed of the protein tyrosine kinase (PTK) C-terminal Src kinase (Csk) and lymphocyte-specific protein kinase (Lck). These kinases phosphorylate the immunoreceptor tyrosine kinase-based activation motifs (ITAMs) on the ζ-chain of CD3. This induces downstream signaling by the recruitment, phosphorylation and activation of the zeta-chain associated protein kinase 70 (ZAP70). ZAP70 initiates a downstream signaling cascade and includes activation of phospholipase Cγ1 (PLCγ1), which promotes calcium mobilization, activation of protein kinase C (PKC) and activation of Ras pathway (30–32). The combination of these signaling cascades promotes activation of several transcription factors, including Nuclear factor kappa-light-chain-enhancer of activated B cells (NFκB), Nuclear factor of activated T-cells (NFAT) and Activator protein 1 (AP-1). This leads to production and secretion of the T cell-specific growth factor IL-2 (33–38). The costimulatory receptor CD28 interacts with its ligands CD80 and CD86, which are differentially expressed by APCs. CD86 is constitutively expressed on APCs, while expression of CD80 is induced by stimulation of Toll-like receptors (TLRs) (39). Activation of CD28 leads to cross phosphorylation of intrinsic CD28 receptor tyrosine residues followed by attachment and activation of phosphatidylinositol kinase 3 (PI3K) (40, 41). PI3K phosphorylates phosphatidylinositol 4,5-bisphosphate (PIP2) to phosphatidylinositol 3,4,5 phosphate (PIP3), leading to activation of protein kinase B (PKB or Akt) and NFκB. This pathway regulates T cell survival through the expression of the anti-apoptotic gene B-cell lymphoma-extra-large (BCL-XL) and Akt-dependent upregulation of IL-2 production (39). CTLA4, however, is considered an inhibitory receptor and stimulation will, to some extent, suppress TCR/CD3-CD28 induced activation by competing with CD28 for CD80/86. Perturbation of the CTLA4 receptor will lead to the recruitment of endogenous protein tyrosine and serine/threonine phosphatases. This includes Src homology protein 2 domain-containing tyrosine phosphatase 2 (SHP-2) and protein phosphatase 2A (PP2A). This will lead to dephosphorylation and inhibition of several proteins in the signaling pathway, including ZAP70, consequently reducing the activation, growth and clonal expansion of T cells (42–47). Exhausted T cells (see below for an explanation) may be found in the environment of chronic infections and cancer cells often display increased expression of inhibitory receptors, including CTLA4 and programmed death receptor 1 (PD-1) (45, 48).

Naïve T cells have relatively low metabolic activity (49), but in response to activating stimuli, metabolic activity is rapidly increased (50). This rapid response is possible due to increased nutrient uptake post stimulation. This may occur concomitant with the presence of untranslated mRNA, idle ribosomes, rapid turnover of transcription factors required for T cell activation, and the presence of an abundance of most of the glycolytic enzymes in quiescent T cells. Together this facilitates upregulation of protein, DNA and lipid synthesis (51, 52). Perturbation of the TCR/CD3 complex and the CD28 marker is further associated with activation of Calcium Calmodulin-dependent protein kinase 2 (CaMKK2) (53). CaMKK2 is known to activate the energy sensor AMP-dependent protein kinase (AMPK) in T cells (54, 55). AMPK is an energy sensor, which is mainly activated by low levels of ATP and liver kinase B1 (LKB1)-dependent phosphorylation (56). Although AMPK is commonly activated in response to an increased ADP/ATP ratio, TCR/CD3-CD28 signaling increases mitochondrial biogenesis and activation of this energy associated enzyme independently of the ADP/ATP ratio (55). This suggests that AMPK activation and increased mitochondrial biogenesis is induced in preparation for the energy demands required for T cell growth and proliferation. Engagement of TCR/CD3-CD28 also promotes recruitment of PI3K and activation of Akt in the immune synapse. This regulates the activity of the mammalian target of rapamycin, mTOR/raptor complex 1 (mTOR complex 1/mTORC1) through inhibition of the key upstream regulator tuberous sclerosis complex 2 (TSC2), which functions as a GTPase activity protein (GAP) for Ras homolog enriched in brain (Rheb) GTPase (57). mTOR kinase forms two distinct protein complexes, mTORC1 and mTORC2, which are crucial for driving differentiation of CD8+ and CD4+ T cells (58). The mTORC1 complex is defined as regulatory-associated protein of mTOR (RAPTOR), while mTORC2 is defined as rapamycin insensitive companion of mTOR (RICTOR) (57).

**Figure 1:**
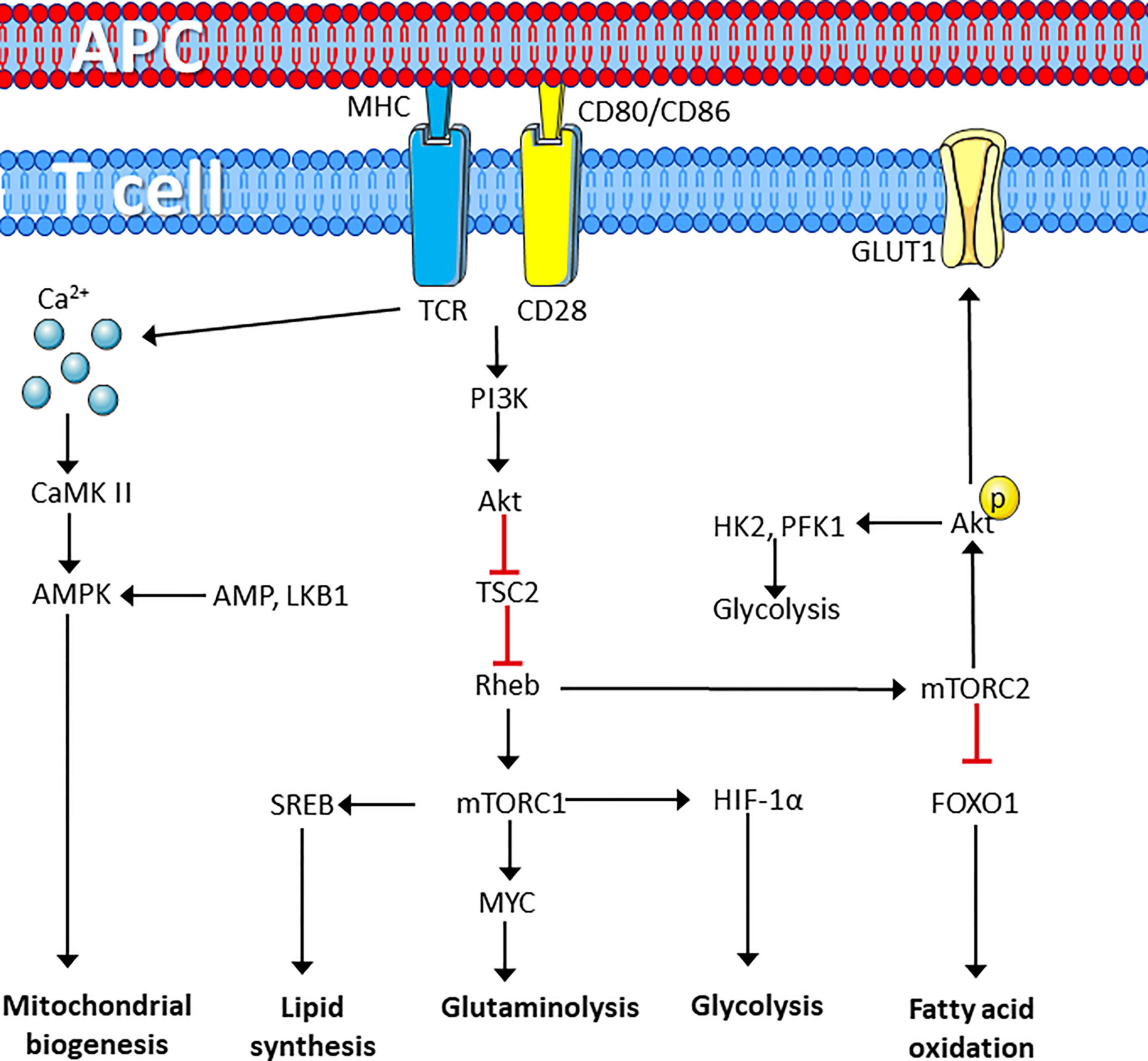
T cell receptor perturbation drives metabolic reprograming. T cell activation by ligation of the TCR and CD28 cell surface marker induces metabolic reprograming through activation of protein kinases that induces calcium dependent activation of the AMPK pathway to induce synthesis of mitochondria. In addition, mTORC1 activation induces lipid synthesis and enhances lipid synthesis, glutaminolysis and glycolysis required for rapid proliferation, whereas mTORC2 induces glucose uptake and glycolysis while repressing fatty acid oxidation. Figure was created using assets from Servier Medical Art, licensed under a Creative Common Attribution 3.0 Generic License. (http://smart.servier.com/). APC, antigen presenting cell; TCR, T cell receptor; PI3K, phosphatidylinositol kinase 3; CaMK II, calcium calmodulin-dependent kinase II; AMPK, adenosine monophosphate-dependent kinase; TSC2, tuberous sclerosis complex 2; Rheb, RAS homolog enriched in the brain; LKB1, liver kinase B1; SREB, sterol regulatory element-binding proteins; HIF-1α, hypoxia-inducible factor 1 α; mTORC, Mechanistic target of rapamycin complex; GLUT1, Glucose transporter 1; FOXO1, forkhead box O1; FAO, fatty acid oxidation; HK1, hexokinase 1; PFK-1, phosphofructokinase 1.

The GTP-bound form of Rheb directly interacts with mTORC1, which tunes the induction and activity of several transcription factors involved in regulation of mitochondrial activity and biomass production. This includes sterol regulatory element-binding proteins (SREBP) hypoxia-inducible factor 1α (HIF-1α) and MYC, resulting in increased glycolysis, glutaminolysis and lipid synthesis (59). mTORC2, on the other hand, is more involved in fatty acid oxidation and negatively regulates CD8+ T cell memory differentiation through Akt-dependent phosphorylation of forkhead box protein O1 (FOXO1) resulting in cytosolic retention (60, 61). mTORC2 directly phosphorylates Akt on Serine 473, thereby, promoting the expression of glucose transporter 1 (GLUT1), activating hexokinase 2 (HK-2) and phosphofructokinase-1 (PFK-1), phosphorylation and expression of 2-phosphofructkinase 6/fructose 2,6-bisphosphatase (PFKFB)-3 and 4. This promotes in increased glucose uptake and glycolysis (summarized in **Figure 1**) (62–65). TCR/CD3-CD28 stimulation is further associated with induction of lactate-dehydrogenase A (LDHA) that converts pyruvate to lactate (66). This metabolic reprograming of T cells result in increased glycolysis and lactate production despite presence of oxygen (67). This process is commonly referred to as the Warburg effect and supports accumulation of glycolytic intermediates that can enter the pentose phosphate pathway (PPP), to produce ribose 5-phosphate (R5P), which is used in nucleotide synthesis. The Warburg effect is also associated with the production of nicotinamide adenine dinucleotide phosphate (NADPH), which has two main functions; it acts as a redox agent and is essential as an electron donor in anabolic biomass synthesis (67, 68). TCR/CD3 and CD4/CD8-induced PTK activity phosphorylates the muscle form of pyruvate kinase (PKM2) leading to nuclear localization of PKM2 dimers, which participate in regulating gene expression rather than glycolysis (69). In fact, regulation of PKM2 activity is a key factor together with pyruvate dehydrogenase kinase 1 (PDK1) in preventing pyruvate entering the mitochondrion. PDK1 inhibits the enzyme pyruvate dehydrogenase (PDH), which catalyzes the formation of acetyl co-enzyme A (AcCoA) and TCA cycle entry. This leads to accumulation of pyruvate in the cytosol, which is used to produce lactate and regenerate NAD+ from NADH (70, 71).

Many proliferating cells, including TCR/CD3-CD28 stimulated T cells, may adopt profiles associated with oxidative phosphorylation (OXPHOS) when glucose is limited. This is achieved by increased uptake and metabolism of the amino acid glutamine (72). This requires upregulation of the glutamine transporter, alanine-serine-cysteine transporter 2 (ASCT2/SLC1A5) and differential regulation of the glutaminase (GLS) isoforms, kidney glutaminase (KGA) and glutaminase C (GAC) (73, 74). TCR/CD3-CD28 co-stimulation upregulates expression of GAC, while downregulating KGA. GLS catalyzes the deamidation of glutamine to glutamate, which is the first step of glutaminolysis. Glutaminolysis is mainly regulated through MYC-induced expression of key genes (75). Glutamine metabolism is crucial for proliferating cells as it is involved in several metabolic pathways. This includes production of α-ketoglutarate (α-KG) from glutamate and alanine by transferring the amino group from glutamate to pyruvate by alanine aminotransferase (ALT) (76). The product, α-KG may enter the TCA cycle to form citrate. Citrate can be shunted out of the mitochondrion to support cytosolic AcCoA levels and used as a substrate lipogenesis and mevalonate metabolism as well as cholesterol synthesis (77, 78). α-KG may also support production of carbon intermediates in the TCA cycle and the production of reduced forms of the electron donors NADH and flavin adenosine dinucleotide (FADH) required for OXPHOS (79). Glutaminolysis also supports production of NADPH through the PPP and TCA cycle, which along with glutamate and cysteine, is used to produce the antioxidant glutathione (GSH) (80, 81). Finally, glutaminolysis provides both carbon and nitrogen residues used for synthesis of polyamines, amino acids and DNA and RNA nucleotides (82). Inhibition of glutaminolysis as well as reduced ornithine and putrescine synthesis decrease T cell proliferation, highlighting the important role of glutaminolysis (83).

As glutamine has a central role in proliferating cells, including activated T cells, glutamine is sometimes referred to as a conditionally essential amino acid. In fact, exogenous glutamine deprivation and inhibition of glutaminolysis prevent T cell activation, proliferation and clonal expansion (84). This reliance on glutamine metabolism is sometimes referred to as “glutamine-addition”. Glutamine-dependent anapleurosis is further known to dictate glucose uptake and glycolysis in proliferating cancer cells (85, 86). Extracellular glutamine depletion is also associated with metabolic reprogramming, as glutamine synthetase (GS) converts glutamate to glutamine, is upregulated (87). Glutamine anapleurosis can also be used to produce the amino acid asparagine, which in addition to being important for protein synthesis, is also regulating activation of T cells by directly enhancing Lck activity (88–90). In response to glutamine deprivation, asparagine becomes an essential amino acid. Despite this, mammals do not possess functional asparaginase, the enzyme required for asparagine to re-enter the TCA cycle (89). In fact, ectopic expression of the catalytically active asparaginase inhibits cell growth and proliferation by redirecting asparagine to the TCA cycle rather than protein synthesis (89, 91). Arginine is another amino acid important for T cell activation, and supplementation of extracellular arginine increases long-term survival and effector functions of T cells, accompanied by a reduction in glycolysis and enhanced oxidative phosphorylation (92). T cell activation and clonal expansion also require uptake of the amino acids cysteine, methionine and the branched chain amino acid (BCAA) leucine. While naïve T cells do not express transporters for cysteine and cystine, this is rapidly induced by CD3/CD28-induced activation. In early activation, uptake of cysteine and methionine is crucial for proliferating T cells (93). Leucine is important for determining the fate of CD4+ T cells as it regulates activation of mTORC1. During leucine depletion the leucine sensor sestrin 2 (SESN2) binds to the GTPase-activating protein towards Rags-2 (GATOR2), forming an inhibitory complex towards mTORC1 (94, 95). Loss of the leucine transporter SLC7A5/LAT1 (CD98) limits T cell activation and effector maturation owing to impairments in mTORC1 activity (96). Finally, serine is used for one-carbon metabolism and purine synthesis through the B vitamin 10-formyltetrahydrofolate. Serine also supports 5-methyltetrahydrofolate, and generation of the methyl donor S-adenosyl methionine (97).

In addition to nutrient availability, oxygen tension also varies greatly in different tissues. Many lymphoid organs, including spleen and thymus, are known to have low oxygen levels and are considered to be in a state of physiological hypoxia (<4% O_2_) (98). T cells, which are highly mobile in nature encounters a wide range of oxygen levels in the body. For example, during thymic development, thymocytes inhabit within relatively low oxygen (< 1%). Activated T cells are faced with both high oxygen levels in the lungs and arterial blood as well as the hypoxic and anoxic conditions in inflammatory lesions and tumors (99–101). As low oxygen concentration is metabolically challenging, hypoxia induces metabolic programs required to adapt to the environment. T cell differentiation, function and survival is known to be affected by exposure to hypoxia, a response mainly mediated by the transcription factor HIF-1α, directly or indirectly (98). HIF-1α, is stabilized and heterodimerizes with the constitutively expressed HIF-1β, also known as aryl hydrocarbon receptor nuclear transporter (Arnt) under hypoxic conditions. The HIF complex translocate into the nucleus where it binds to hypoxia response elements (HREs) (102). In the presence of oxygen, HIF-1α is rapidly degraded by the enzyme prolyl hydroxylase domain proteins (PHD) 1, 2 and 3, thus the HIF-1α complex has limited transcriptional activation capacity in normoxia. HIF-1α induces a metabolic shift by inducing a glycolytic phenotype mediated by increased expression of genes involved in glucose uptake and glycolysis, including GLUT1, HK2, PKM2, LDHA, while actively reducing glucose oxidation through the expression of PDK1 (102–104). This indicates that HIF-1α induces a metabolic phenotype where glycolysis is the primary source for ATP. As pyruvate is shunted towards lactate, rather than the TCA cycle, this reduces the ability to produce citrate. To compensate for this, cells at hypoxia produce citrate and cytosolic AcCoA required for fatty acid synthesis through glutaminolysis. In this case, α-KG is metabolized to citrate through the TCA cycle in a reverse fashion by reductive carboxylation. These reactions are catabolized by the two isocitrate dehydrogenase (IDH) isozymes, IDH1 and IDH2, which are induced by low oxygen (105, 106). This demonstrates that during hypoxia, HIF-1α drives T cells to adapt a metabolic phenotype relying on glycolysis and glutaminolysis to support proliferation and clonal expansion.

**Figure 2:**
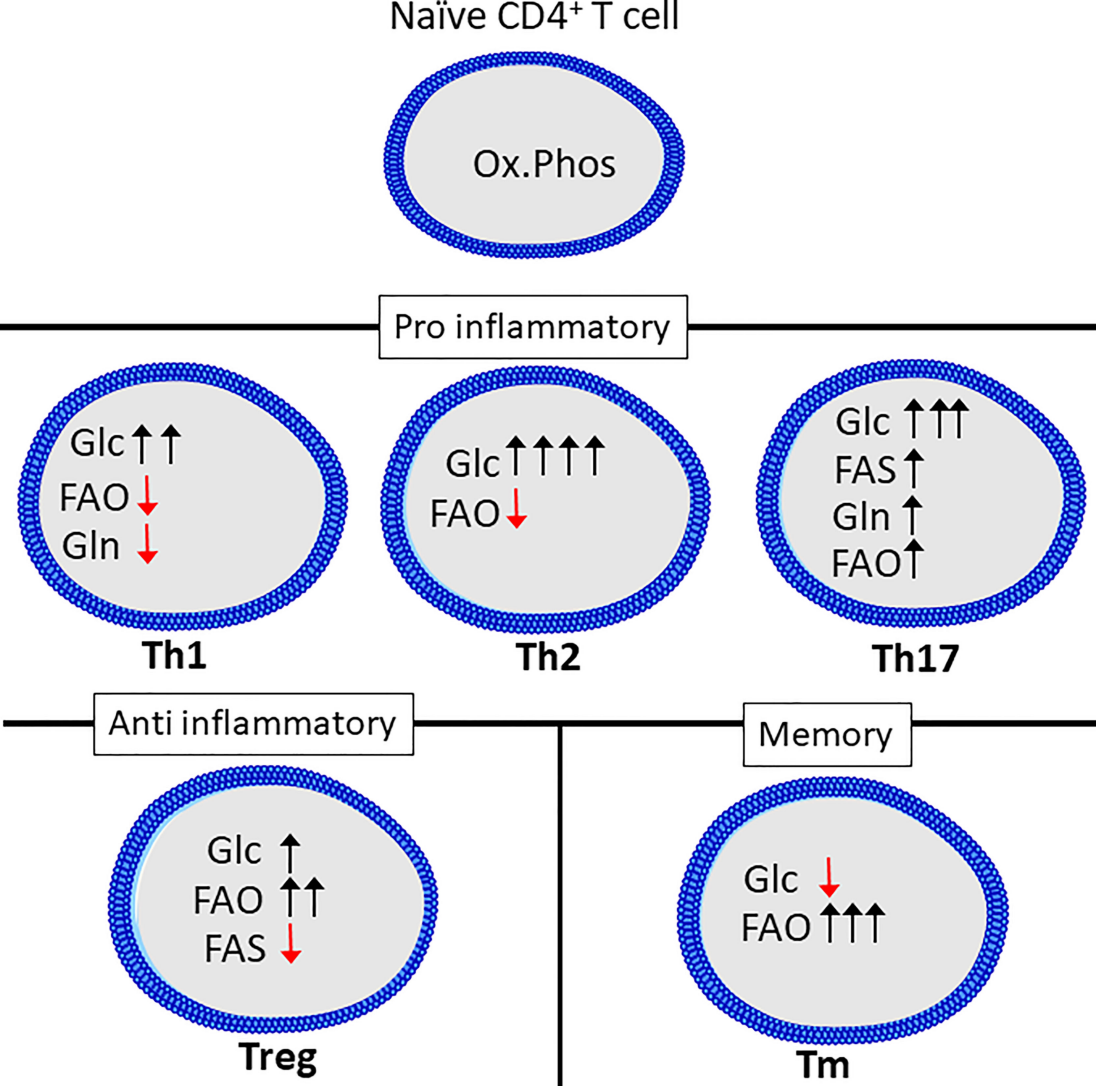
Th subsets display distinct metabolic programs. Quiescent Th cells rely mainly on oxidative phosphorylation (Ox.Phos) to maintain homeostasis, while the effector subsets are characterized by increased glycolytic metabolism (Glc) as well as a differential reliance on glutaminolysis (Gln), fatty acid oxidation (FAO and fatty acid synthesis (FAS) to support effector functions. Figure was created using assets from Servier Medical Art, licensed under a Creative Common Attribution 3.0 Generic License. (http://smart.servier.com/t).

In addition to its role in early activation, it has become increasingly clear that distinct metabolic programs define the various T cell subsets (**Figure 2**) (107). It is also known that T cell differentiation can be manipulated through modulating metabolic activity *in vitro* (108–110). However, the extent of how metabolism affects T cell function in response to infection is not fully understood.

As mentioned, naïve T cells generate most of their ATP from oxidative phosphorylation and in general display a less energetic state than activated T cells yet can rapidly reprogram metabolism upon activation (50, 111, 112). This rapid response towards antigen is likely made possible by the rapid turnover of proteins (51). While this metabolic reprograming stems, at least in part, from an increased demand for ATP to fuel rapid proliferation, there is also increasing evidence that fine-tuning of specific metabolic pathways drive differentiation into various subsets. This is best characterized for the Th1, Th2 and Th17 subsets.

Th1, Th2 and Th17 cell differentiation induces three distinct metabolic phenotypes. However, all subsets have a relatively high rate of glycolysis when compared to Tregs, naïve or memory T cells (113). While Glucose is known to be crucial for T cells to produce IFNγ, as cells cultured in galactose have severely reduced IFNγ-secretion. This has been linked to the dual role of the enzyme glyceraldehyde 3-phosphate dehydrogenase (GAPDH), as it can bind to, and repress translation of IFNγ mRNA (110). This was shown to be related to glucose-derived mannose combustion. Supplementation with mannose could partially restore IFNγ production in cells cultured with the glycolysis inhibitor 2-deoxy-D-glucose (2DG). Interestingly, the transcription factor T-bet, which is crucial for Th1 differentiation, was reduced when cells were cultured in the presence of galactose, but was restored by mannose supplementation (114). Lastly, inhibition of GLS-1 has been shown to increase Th1 differentiation (73). Th2 cells are reportedly the most glycolytic of the Th subsets, correlating with high expression of GLUT1 and low rate of fatty acid oxidation (113). mTORC2, which as previously mentioned, is an important regulator of fatty acid oxidation and glycolysis, is also important for differentiation of Th1 and Th2 cells through distinct mechanisms. Th1 cells depend on mTORC2-dependent phosphorylation of Akt, while Th2 cells depend on phosphorylation of PKC (115). Peroxisome proliferation activating receptor-γ (PPAR-γ), which is a regulator of fatty acid metabolism, is also involved in promoting Th2 differentiation while repressing Th1 differentiation (116). Th17 differentiation induces a metabolic phenotype distinct from both Th1 and Th2 cells. Th17 differentiation requires HIF-1α stabilization and induction of PDHK1, preventing mitochondrial pyruvate oxidation in favor of lactate production (71, 104, 117). GLS1-dependent glutamine metabolism is also a crucial factor for Th17 differentiation (109). GLS1 expression in T cells can be induced by the transcription factor inducible cAMP early repressor (ICER) (109). Blocking GLS1 shifts Th17 cells to a Th1-like phenotype (73). Th17 differentiation also requires fatty acid synthesis (118). The metabolic phenotype of Th9 and Th22 cells is not as extensively studied compared to that of the other CD4+ effector cells. However, Th9 cells have a higher glycolytic rate than both Th2 and Th17 cells, and require activation of mTORC1 and HIF-1α (119).

Treg differentiation depends on the oxidation of long fatty acids, induced by activation of AMPK (113). Although Tregs upregulate glycolysis compared to naïve cells this is to a lesser degree than the effector CD4+ T cells and it is not necessary to support Treg differentiation (113, 120). In support of this Treg differentiation is inhibited in favor of Th17 differentiation by HIF-1α-induced glycolysis (117, 121). In fact, increasing glucose uptake and glycolysis in Tregs represses suppressor functions despite increasing proliferation (122, 123). Compared to effector CD4+ T cells, Tregs depend on fatty acid oxidation to fuel mitochondrial respiration (120). Treg differentiation may also be reduced by the presence of exogenous amino acids including glutamine, tryptophan and arginine (124). Lastly, extracellular lactate may enhance Treg function through uptake *via* the monocarboxylate transporter 1 (MCT1) (123).

Memory subsets of T cells are characterized by a reliance on fatty acid oxidation and low rate of glycolysis (125). Supplementing the amino acid arginine has been shown to enhance memory formation and reprogram T cells from a glycolytic to an oxidative phenotype (92). Similar to naïve T cells, memory T cells have a rapid turnover of proteins related to activation, including glycolytic enzymes (51).

The microenvironment is also important for modulating T cell activity and metabolism. Recently, it was discovered that T cells create acidic niches within lymphoid organs to dampen effector functions (126). This is also well-defined in the tumor microenvironment (TME), which is characterized by the presence of anti-inflammatory cytokines, low levels of oxygen and acidification by lactate (127–130). In rheumatoid arthritis the microenvironment of the synovium exerts pro-inflammatory effects on the CD4+ T cells by repressing glycolytic rate (131). It is further known that increased body temperature may enhance proliferation and effector functions of both CD4+ and CD8+ T cells (132, 133) Together this demonstrates how the microenvironment may shape T cell activity.

T cell exhaustion is featured in both cancer and persistent infections. Exhausted T cells can be defined by an increased expression of inhibitory ligands, including PD-1 and CTLA-4 and are more apoptotic than effector and memory cells (48, 134). Exhausted T cells also display a distinct metabolic phenotype, with reduced glucose uptake as well as mitochondrial dysfunction, which reduces capacity for oxidative metabolism. The presence of extracellular metabolites as well as low abundance of important nutrients may further inhibit metabolism and prevent the appropriate T cell activity (135, 136).

## T Cell Metabolism in Infection

As detailed above, T cell metabolism and functionality are closely related and metabolic reprograming is crucial for appropriate T cell activity. In this section, we will briefly describe how T cell metabolism is regulated in certain acute infections and how T cell metabolism may aid or hinder pathogen clearance under acute and chronic conditions of infection. We will use examples of virus infections by SARS-CoV-2 and HIV as these viruses are known to induce differential effects on T cell metabolism. We will also briefly describe T cell metabolism in bacterial infections, here exemplified by infections of Mycobacterium tuberculosis.

### T Cell Metabolism in SARS-CoV-2 Infections

When T cells are engaged in the immune response, they can be roughly divided into naïve, effector and memory cells, each with accompanying metabolic programs (137). The immune system, when properly regulated will have a protective role. However, in some cases the immune system might exasperate the inflammation associated with infection (138). This is apparent for SARS-CoV-2 infections, which cause the well-known coronavirus disease 2019 (COVID-19). Despite that the underlying molecular mechanism responsible for sustaining SARS-CoV-2 virulence is enigmatic, how SARS-CoV-2 attaches on the surface of host cells through a variety of receptors is in part well described. Attachment may be through receptors, such as angiotensin converting enzyme 2 (ACE2), neuropilin-1 (NRP1), AXL, and antibody–FcγR complexes (139). ACE2 is expressed in various human organs and may play a role in regulating cardiovascular and renal function. In addition, the ACE2 protein is a functional receptor for the spike glycoprotein of the human coronavirus and is considered a causative agent of COVID-19 disease. Next, NRP1 is a membrane-bound coreceptor to a tyrosine kinase for both vascular endothelial growth factor (VEGF) and semaphorin (SEMA3A) family members, both playing versatile roles in angiogenesis, axon guidance, cell survival, migration, and invasion. Furthermore, the gene AXL, which encodes the tyrosine-protein kinase receptor UFO, is involved in stimulation of cell proliferation and survival through PI3K-AKT-mTOR, MEK/ERK, NF-κB, and JAK/STAT activation (140–143). In line with the fact that COVID-19 uses receptor ligation for infection, several reports reveal that COVID-19 ligation through and activation of the above-mentioned signal transduction molecules, induces metabolic reprogramming (144, 145). In fact, this metabolic reprograming can be detected by positron emission tomography (PET) by increased accumulation of ^18^F labelled fluorodeoxyglucose (FDG) (146). This metabolic reprograming is also associated with a distinct profile of serum metabolites, which might be used as a prognostic measurement of disease severity (147). It has further been demonstrated that this increased level of FDG in the tissue correlate with increased glycolysis in several cells, including epithelial cells and immune cells, reviewed by Kumar (148). Notably, peripheral blood mononuclear cells (PBMCs) display metabolic dysfunction, characterized by increased glycolysis and reduced oxygen consumption (149). SARS-CoV 2-infected monocytes also show enhanced glycolytic rate. Interestingly, the same study reports that increasing extracellular glucose concentration increased SARS-CoV 2 replication in monocytes (150). Because of this central role of glycolysis in COVID-19 the glucose analog 2-deoxy-D-glucose (2DG), which inhibits glycolysis, has undergone a phase III trial and received emergency approval in the treatment of moderate and severe COVID-19 in India. However, the trial was conducted in only 220 patients and the data has not been made available to the public (151). In this review we have mainly focused on the metabolism of T cells in response in COVID-19.

**Figure 3:**
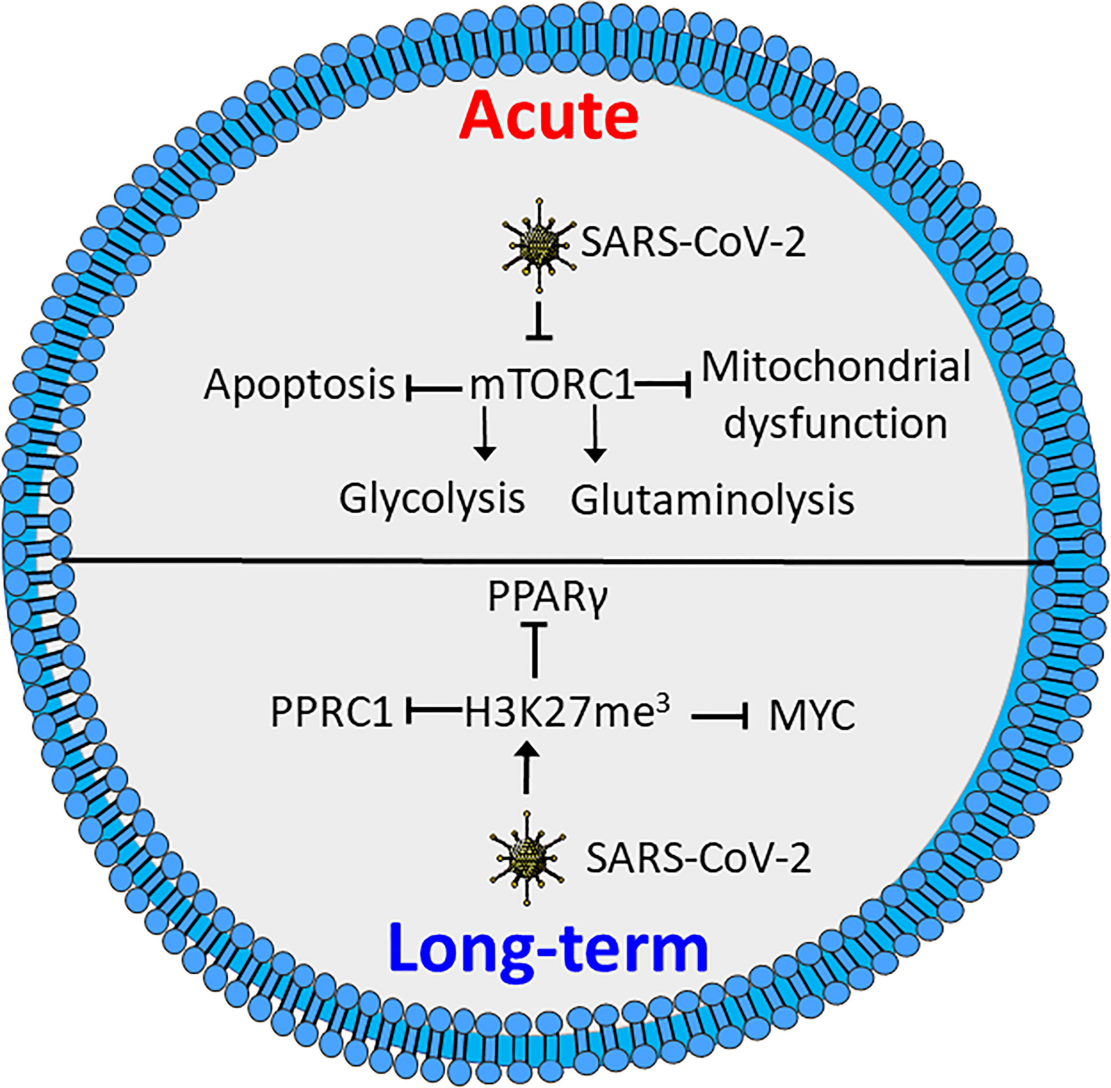
SARS-CoV-2 induces metabolic defects in acute and long-term infection. Acute infection by SARS-CoV-2 inhibits activation of mTORC1, causing reduced glycolysis and glutaminolysis as well as enhanced apoptosis and mitochondrial defects. Long-term SARS-CoV-2 induces triple methylation of histone 3 lysine 27 (H3K27me^3^), resulting in reduced activation through inhibition of MYC peroxisome proliferator-activated receptor γ (PPARγ) and peroxisome proliferator-activated receptor gamma coactivator-related protein 1 (PPRC1). Figure was created using assets from Servier Medical Art, licensed under a Creative Common Attribution 3.0 Generic License. (http://smart.servier.com/).

In both CD4+ and CD8+ T cells COVID-19 inhibits activation of mTORC1, which reduces glycolytic activity, as well as causing mitochondrial dysfunction and increased susceptibility to apoptosis (**Figure 3**) (145). In line with this, expression levels of GLUT1 are reported to be decreased in T cells in patients with severe COVID-19 as compared to healthy controls or patients infected by influenza virus. However, contradictory results exist. A study by De Biasi et al., showed with one exception that T cells from COVID-19 patients had a similar capacity for metabolic reprogramming to non-infected T cells (152). However, the COVID-19 patients all required respiratory aid, while most patients in the latter study had pO_2_ >90%. This might indicate a link between metabolic alteration, mitochondrial dysfunction and declining oxygen saturation (145, 152). This is supported by a study by Siaska et al., where T cell metabolism was differentially affected in mild compared to moderate and severe disease. This was evident by an elevated uptake of fatty acids in T cells from patients with mild or no symptoms, while glucose uptake was similar to that of quiescent T cells from healthy controls (153). This study also revealed that moderate and severe disease was correlated with increased mitochondrial content, ROS and expression of basigin, which is reported to drive hyperinflammation and bone degradation in rheumatoid arthritis (153, 154).

Several studies have shown that recovered COVID-19 patients display symptoms for weeks or months after disease onset, commonly referred to as “long COVID” (155–157). These symptoms include chest pains, joint pains, cognitive disorders, anxiety, depression and neurological disorders. Long COVID even affects individuals who initially had mild symptoms upon infection (155, 156, 158). Interestingly, T cells from COVID-19 patients reportedly have increased levels of triple methylated histone 3 lysine 27 (H3K27me^3^), a potentially epigenetic effect that persists in recovered patients (159). Reports demonstrate that the H3K27me^3^ modification represses T cell activation, differentiation and metabolic activity, by inhibiting expression of several transcription factors involved in metabolic regulation. These include MYC, PPARγ and peroxisome proliferator-activated receptor gamma coactivator-related protein 1 (PPRC1) (**Figure 3****)** (160, 161). A recent study demonstrates a correlation between neurologic symptoms in recovered patients and T-cell immune reactivity. Patients with neurologic symptoms were also shown to have a distinct T-cell phenotype compared to healthy COVID convalescents. Notably, in individuals with long COVID Tfh cells had increased reactivity to the nucleocapsid of the SARS-CoV-2 virus, while the COVID convalescents showed reactivity towards spike proteins (157). This is consistent with an earlier study revealing that T cells from patients with neurological symptoms following COVID-19 recovery, displayed a distinct reactivity. The same study also showed an increase in the population of exhausted CD4+ T cells (162). Whether these effects are related to the epigenetic changes induced by methylation of H3K27 is not clear. Further studies will be needed to determine how T-cell metabolism and metabolic dysfunction are correlated to COVID-19 disease severity and if similar phenomena can be found for other infectious agents.

As T-cell metabolism is correlated to both short-term survival and long-term effects further studies of T-cell metabolism in COVID-19 may result in better stratification of patients as well as offering new therapeutic targets to minimize the impact of COVID-19.

### T Cell Metabolism and HIV Infections

**Figure 4:**
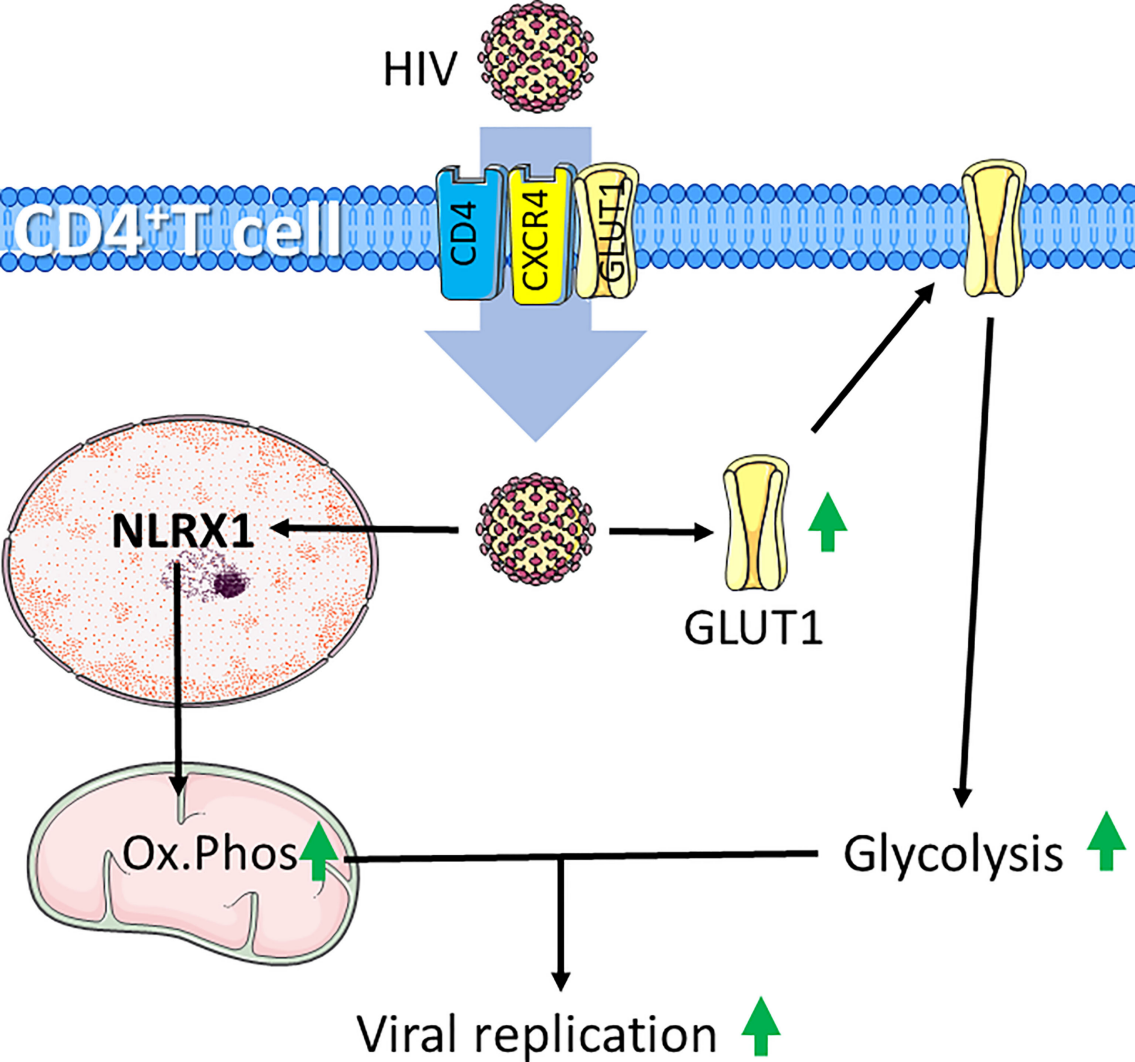
HIV infection induces increased glycolysis and mitochondrial respiration in CD4+ T cells. HIV infects CD4+ T cells through interaction with CD4 and chemokine receptor CXCR4. HIV-infection supports glycolytic metabolism by increasing expression of the glucose transporter GLUT1, which can also be exploited to further infect CD4+ T cells. Additionally, HIV induces expression the of nucleotide-binding domain leucine-rich repeat-containing receptor X1 (NLRX1) to induce increased oxidative phosphorylation (Ox.Phos). The increased metabolic rate supports enhanced viral replication. Figure was created using assets from Servier Medical Art, licensed under a Creative Common Attribution 3.0 Generic License. http://smart.servier.com/.

The excessive immune response for severely affected patients may be caused by so called cytokine storms, which is simply defined as release of too many cytokines into the blood too quickly (163). In patients where this occurs, coupled with inability to clear infection leads to chronic infection, a condition leading to T-cell exhaustion (137). Exhausted T cells are characterized by an increased expression of inhibitory markers and a progressive and hierarchical loss of function as seen in HIV infections, where HIV is the causative agent for acquired immunodeficiency syndrome (AIDS) (164). HIV target the CD4 cell surface marker, allowing infection of CD4+ T cells and Mø (165). During the acute phase of HIV infection, most patients experience a marked increase in HIV viral load coupled with increased levels of the PD‐1 receptor on HIV‐infected T cells. Untreated patients will in this case experience T-cell exhaustion. The positive effects of antiretroviral therapy (ART), which in most cases significantly inhibits viral replication, also lowers high PD-1 expressing T cells, suggesting a link between viral load and the level of PD-1 expressing T cells (166, 167). The HIV virus infects cells by first interacting with CD4, followed by interaction with the chemokine receptor CCR5 on Mø or CXCR4 on T cells (168). In addition to CD4 and CXCR4, HIV is reported to exploit GLUT1 to infect T cells (169). GLUT1 expression has been shown to be elevated on circulating CD4+ T cells in patients with chronic HIV-1 infection. As HIV-induced surface expression of GLUT1 coincide with increased glucose uptake and increased glycolytic activity (**Figure 4****)** (170, 171) it is likely that such a metabolic pattern mirror HIV infected CD4+ T cells in AIDS patients. Recently, it was demonstrated that in acute HIV-1 infections, viral replication is correlated with increased expression of nucleotide-binding domain leucine-rich repeat-containing receptor X1 (NLRX1). NLRX1 is most likely required for the HIV-induced metabolic reprograming of CD4+ T cells and leads to increased glycolysis and oxygen consumption. Interestingly, blocking glycolysis using the glucose analogue 2DG or mitochondrial respiration using rotenone or metformin inhibits HIV replication in CD4+ cells (171). This is in line with the fact that CD4+ T cells cultured with the simple sugar galactose exhibit less viral replication and reduced HIV-induced cell death, supporting that HIV infections requires glycolytic activity (172). Furthermore, some HIV-infected patients maintain low virus titers even without ART. This was associated with distinct HIV-specific CD8+ T cells. Such CD8+ T cells can be distinguished from CD8+ T cells from patients sensitive to ART. In the ART-insensitive patients, the CD8+ T cells expressed enhanced levels of mTORC2 and increased fatty acid oxidation. and ART-insensitive patients were further associated with formation of increased numbers of CD8+ memory T cells Interestingly, in ART treated CD8+ T cells, treatment with IL15 reflected increased mTORC2 expression and a fatty acid consuming phenotype (173). In line with these observations, T-cell metabolism may have prognostic potential, and may even provide therapeutic targets in cases of HIV infections.

The current knowledge of T-cell metabolism in HIV infection indicates that the HIV virus exploits the glycolysis of CD4+ T cells to both infect and enhance replication rate, while high glycolytic rate of CD8+ T cells confer resistance to infection (170, 171, 173). Hence, both CD4+ and CD8+ T-cell metabolic profiles may serve as prognostic markers of the patients. In line with this, it is speculated that targeting glycolysis of e.g., CD4+ T cells may reduce T-cell replication in patients suffering from viral infection, such as by HIV. Importantly, this understanding of metabolism in HIV indicates a potential for repurposing metformin for the management of HIV infection (171). Together, this highlights the importance of further studies to determine the efficacy of metabolic inhibition in the management of HIV infection.

### T-Cell Metabolism in Tuberculosis Infections

Tuberculosis remains a major threat to human global health, with an estimated one-fourth of the world population latently infected (174). Tuberculosis is caused by *Mycobacterium tuberculosis* (MT) infection. T cells and Mø are crucial players in the anti-mycobacterial host defense and in containing the spread of mycobacteria during latent disease (175). This is highlighted by the observation that HIV-induced depletion of CD4+ T cells and the level of PD-1 expression leads to increased co-morbidity in MT-infected individuals (176, 177). Together this points to the importance of stringent regulation of CD4+ T cells in defense against MT infection.

With an increasing body of evidence, it is argued that metabolic changes at the cellular level, is vital to mount an effective immune response to MT. This include pathways found to be necessary for the full activation of lymphocytes and include regulation of cytokine production, pyrimidine metabolism, as well as production of glutathione and large amounts of mitochondrial reactive oxygen species (ROS) in response to MT infection (178). To this end, the mitochondrial matrix protein Cyclophilin D (CypD) acts as a peptidyl-prolyl cis-trans isomerase that regulates the mitochondrial permeability transition pore (PTP). PTP is a nonspecific large conductance pore when open leads to cell death. PTP expression has been implicated in ischemia/reperfusion injury in multiple organs, in neurodegenerative disorders, and in muscular dystrophies (179, 180). Interestingly, loss of CypD is implicated in metabolic changes, which include increased aerobic glycolysis, mitochondrial OXPHOS, and consumption of glucose and glutamine. This is further associated with increased T-cell activation to proliferation and cytokine production, such as TNFα and IFNγ. Moreover, MT infection in CypD ablated individuals, lead to immunopathology caused by T-cell dysfunction, however, without affecting bacterial burden. Moreover, CypD-deficient mice succumbed earlier to infection than wild-type mice stressing the role of CypD and dysregulated T-cell function potentially associated with altered immune cell metabolism in MT infections (181). It is also reported that both early and long-term MT infection induces distinct defects to the metabolism of CD8+ T cells. MT infection increases expression of both PD-1 and CTLA4, which further correlates with decreased glucose uptake as well as reduced glycolysis and mitochondrial respiration. This further reflects a repressed metabolic programing normally induced by stimulation of the anti-CD3/CD28 complex (182).

**Figure 5:**
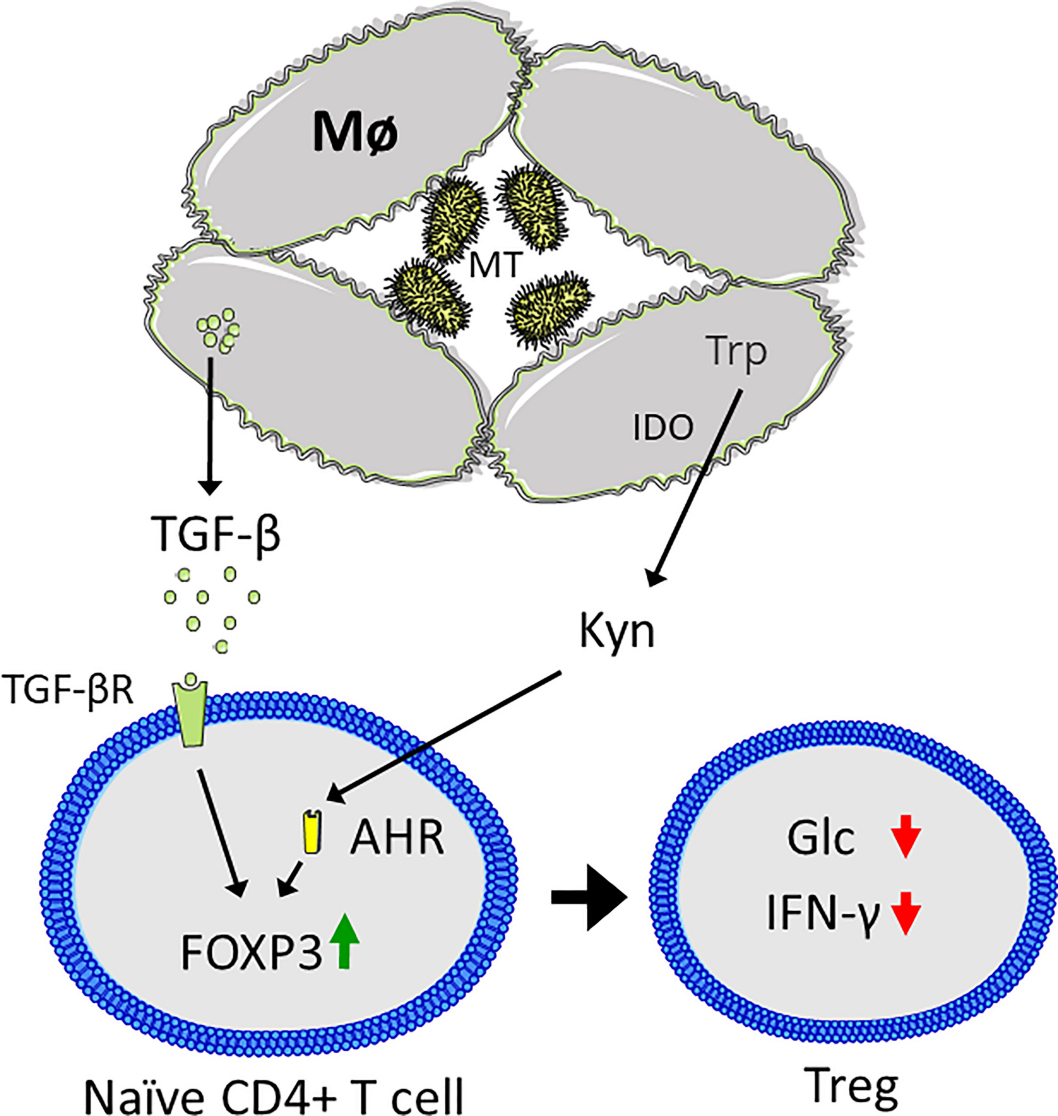
*Mycobacterium tuberculosis* (MT) infection results in an immunosuppressive microenvironment. Macrophages (Mø) are unable to clear MT infection, resulting in granuloma formation. MT induces expression of indoleamine 2,3-dioxygenase (IDO), which catabolized tryptophan (Trp) to kynurenine (Kyn), which binds to the aryl hydrocarbon receptor (AHR) in CD4+ T cells, coupled with secretion of transforming growth factor β (TGF-β) that binds to the TGF-β receptor (TGF-βR). Together this results in upregulation of FOXP3 and Treg differentiation, characterized by suppression of CD4+ T cell glycolysis (Glc) and interferon γ (IFN-γ) secretion. Figure was created using assets from Servier Medical Art, licensed under a Creative Common Attribution 3.0 Generic License. (http://smart.servier.com/).

As the tissue microenvironment also affects the effector functions and metabolism of T cells, targeting metabolic pathways can also affect T-cell activity indirectly. This is the case for MT-induced granulomas, in which Mø catabolize tryptophan and secrete transforming growth factor β (TGF-β) to create an immunosuppressive environment (178, 183, 184). Hence, the capability of T cells in clearing MT infections is largely limited by the inability to enter the granulomas in which MT induces expression of indoleamine 2,3-dioxygenase (IDO), and tryptophan catabolism to form the immunosuppressive metabolite kynurenine (185, 186). Moreover, IDO expression has previously been linked to a decline in IFNγ, demonstrating how MT-induced changes in Mø metabolism may induce tolerance and prevent clearance (183). This likely occurs through interaction between kynurenine and the aryl hydrocarbon receptor (AHR) in combination with TGF-β, which drives Treg differentiation (**Figure 5**) (187). In conclusion, MT infections contribute to decreased T cell glycolytic metabolism through several mechanisms. Despite that the knowledge is sparse, such changes may further have implications for how and to what extent the immune system is able to counteract a MT infection and to what extent the immune system may be capable to eradicate the MT bacteria.

## Conclusion

We have briefly summarized some knowledge on T-cell metabolism in situation of infections by virus and bacteria. Research over the last couple of decades have revealed that T-cell metabolism is closely linked, not only to proliferation and clonal expansion, but that cell-specific metabolism also dictate cell differentiation programs and reflect effector functions (136). As detailed here, T-cell metabolism may in addition aid pathogen clearance or confer resistance to infections by virus and bacteria. However, in some cases T-cell metabolism may be displayed as T-cell exhaustion leading to tolerance, or even support of inflammation. The latter is displayed in certain cases of viral infection, where immune cell metabolism also supports viral replication (135, 169, 170, 181, 182).

Modulating T-cell metabolism by supplementation with key nutrients or employing inhibitors of key metabolic pathways has previously been shown to enhance memory formation or enhance effector function of T cells in cancer, or even induce pro-inflammatory and migratory programs (92, 108, 188, 189). As T-cell exhaustion is a feature in both persistent infection and cancer it is expected that reversing the exhausted phenotype will have markedly clinical potential (190). T-cell metabolism may also be important in both the acute and long-term effects of COVID-19, making it an interesting target for therapeutic applications (145, 155, 159, 162).

As detailed in this review, metabolic reprograming of T cells is crucial for conveying protection against infectious pathogens. On the other hand, some pathogens may exploit or modify T cell metabolism to prevent the appropriate effector functions. Further research is needed to determine if targeting dysfunctional T cell metabolism may be used therapeutically in infectious diseases to enhance effector functions and reverse T-cell exhaustion in chronic infections.

## Author Contributions

JW and BS both conceived and drafted this review and contributed to final proof and approval of the submitted version.

## Funding

Grant from Thorn Holst 2020-2021 to BS.

## Conflict of Interest

The authors declare that the research was conducted in the absence of any commercial or financial relationships that could be construed as a potential conflict of interest.

## Publisher’s Note

All claims expressed in this article are solely those of the authors and do not necessarily represent those of their affiliated organizations, or those of the publisher, the editors and the reviewers. Any product that may be evaluated in this article, or claim that may be made by its manufacturer, is not guaranteed or endorsed by the publisher.

## References

[1] Coates M Lee MJ Norton D MacLeod AS. The Skin and Intestinal Microbiota and Their Specific Innate Immune Systems. Front Immunol (2019) 10:2950. doi: 10.3389/fimmu.2019.02950 31921196 PMC6928192

[2] Murphy K Weaver C Janeway C. Janeway’s Immunobiology. New York: Garland Publishing Inc. (2017).

[3] Medzhitov R. Recognition of Microorganisms and Activation of the Immune Response. Nature (2007) 449(7164):819–26. doi: 10.1038/nature06246 17943118

[4] Tang D Kang R Coyne CB Zeh HJ Lotze MT. PAMPs and DAMPs: Signal 0s That Spur Autophagy and Immunity. Immunol Rev (2012) 249(1):158–75. doi: 10.1111/j.1600-065X.2012.01146.x PMC3662247 22889221

[5] Alberts B. Molecular Biology of the Cell. 5th Edition. New York: Garland Science (2008).

[6] Schmidt ME Varga SM. The CD8 T Cell Response to Respiratory Virus Infections. Front Immunol (2018) 9:678. doi: 10.3389/fimmu.2018.00678 29686673 PMC5900024

[7] Sutton VR Davis JE Cancilla M Johnstone RW Ruefli AA Sedelies K. Initiation of Apoptosis by Granzyme B Requires Direct Cleavage of Bid, But Not Direct Granzyme B-Mediated Caspase Activation. J Exp Med (2000) 192(10):1403–14. doi: 10.1084/jem.192.10.1403 PMC2193191 11085743

[8] Geginat J Paroni M Maglie S Alfen JS Kastirr I Gruarin P. Plasticity of Human CD4 T Cell Subsets. Front Immunol (2014) 5:630. doi: 10.3389/fimmu.2014.00630 25566245 PMC4267263

[9] Infante-Duarte C Kamradt T. Th1/Th2 Balance in Infection. Springer Semin Immunopathol (1999) 21(3):317–38. doi: 10.1007/BF00812260 10666776

[10] Khader SA Gaffen SL Kolls JK. Th17 Cells at the Crossroads of Innate and Adaptive Immunity Against Infectious Diseases at the Mucosa. Mucosal Immunol (2009) 2(5):403–11. doi: 10.1038/mi.2009.100 PMC2811522 19587639

[11] Ma CS Chew GYJ Simpson N Priyadarshi A Wong M Grimbacher B. Deficiency of Th17 Cells in Hyper IgE Syndrome Due to Mutations in STAT3. J Exp Med (2008) 205(7):1551–7. doi: 10.1084/jem.20080218 PMC2442632 18591410

[12] Capone A Volpe E. Transcriptional Regulators of T Helper 17 Cell Differentiation in Health and Autoimmune Diseases. Front Immunol (2020) 11:348. doi: 10.3389/fimmu.2020.00348 32226427 PMC7080699

[13] Duhen T Geiger R Jarrossay D Lanzavecchia A Sallusto F. Production of Interleukin 22 But Not Interleukin 17 by a Subset of Human Skin-Homing Memory T Cells. Nat Immunol (2009) 10(8):857–63. doi: 10.1038/ni.1767 19578369

[14] Fujita H. The Role of IL-22 and Th22 Cells in Human Skin Diseases. J Dermatol Sci (2013) 72(1):3–8. doi: 10.1016/j.jdermsci.2013.04.028 23746568

[15] Li J Chen S Xiao X Zhao Y Ding W Li XC. IL-9 and Th9 Cells in Health and Diseases-From Tolerance to Immunopathology. Cytokine Growth Factor Rev (2017) 37:47–55. doi: 10.1016/j.cytogfr.2017.07.004 28739029 PMC5632571

[16] Chandwaskar R Awasthi A. Emerging Roles of Th9 Cells as an Anti-Tumor Helper T Cells. Int Rev Immunol (2019) 38(5):204–11. doi: 10.1080/08830185.2019.1648453 31401904

[17] Juno JA van Bockel D Kent SJ Kelleher AD Zaunders JJ Munier CML. Cytotoxic CD4 T Cells—Friend or Foe During Viral Infection? Front Immunol (2017) 8:19. doi: 10.3389/fimmu.2017.00019 28167943 PMC5253382

[18] Tabarkiewicz J Pogoda K Karczmarczyk A Pozarowski P Giannopoulos K. The Role of IL-17 and Th17 Lymphocytes in Autoimmune Diseases. Arch Immunol Ther Exp (2015) 63(6):435–49. doi: 10.1007/s00005-015-0344-z PMC4633446 26062902

[19] Tesmer LA Lundy SK Sarkar S Fox DA. Th17 Cells in Human Disease. Immunol Rev (2008) 223:87–113. doi: 10.1111/j.1600-065X.2008.00628.x 18613831 PMC3299089

[20] Dardalhon V Korn T Kuchroo VK Anderson AC. Role of Th1 and Th17 Cells in Organ-Specific Autoimmunity. J Autoimmun (2008) 31(3):252–6. doi: 10.1016/j.jaut.2008.04.017 PMC3178062 18502610

[21] Romagnani S. Immunologic Influences on Allergy and the TH1/TH2 Balance. J Allergy Clin Immunol (2004) 113(3):395–400. doi: 10.1016/j.jaci.2003.11.025 14758340

[22] Jia L Wu C. The Biology and Functions of Th22 Cells. In: Sun B, editor. T Helper Cell Differentiation and Their Function. Dordrecht: Springer Netherlands (2014). p. 209–30.

[23] Lu Y Wang Q Xue G Bi E Ma X Wang A. Th9 Cells Represent a Unique Subset of CD4(+) T Cells Endowed With the Ability to Eradicate Advanced Tumors. Cancer Cell (2018) 33(6):1048–60.e7. doi: 10.1016/j.ccell.2018.05.004 29894691 PMC6072282

[24] Pennock ND White JT Cross EW Cheney EE Tamburini BA Kedl RM. T Cell Responses: Naïve to Memory and Everything in Between. Adv Physiol Educ (2013) 37(4):273–83. doi: 10.1152/advan.00066.2013 PMC4089090 24292902

[25] Corthay A. How do Regulatory T Cells Work? Scand J Immunol (2009) 70(4):326–36. doi: 10.1111/j.1365-3083.2009.02308.x PMC2784904 19751267

[26] Bantug GR Galluzzi L Kroemer G Hess C. The Spectrum of T Cell Metabolism in Health and Disease. Nat Rev Immunol (2018) 18(1):19–34. doi: 10.1038/nri.2017.99 28944771

[27] Chapman NM Boothby MR Chi H. Metabolic Coordination of T Cell Quiescence and Activation. Nat Rev Immunol (2020) 20(1):55–70. doi: 10.1038/s41577-019-0203-y 31406325

[28] Chapman NM Chi H. Hallmarks of T-Cell Exit From Quiescence. Cancer Immunol Res (2018) 6(5):502–8. doi: 10.1158/2326-6066.CIR-17-0605 29716982

[29] Smith-Garvin JE Koretzky GA Jordan MS. T Cell Activation. Annu Rev Immunol (2009) 27:591–619. doi: 10.1146/annurev.immunol.021908.132706 19132916 PMC2740335

[30] Mariuzza RA Agnihotri P Orban J. The Structural Basis of T-Cell Receptor (TCR) Activation: An Enduring Enigma. J Biol Chem (2020) 295(4):914–25. doi: 10.1016/S0021-9258(17)49904-2 PMC6983839 31848223

[31] Wang H Kadlecek TA Au-Yeung BB Goodfellow HE Hsu LY Freedman TS. ZAP-70: An Essential Kinase in T-Cell Signaling. Cold Spring Harb Perspect Biol (2010) 2(5):a002279. doi: 10.1101/cshperspect.a002279 20452964 PMC2857167

[32] Williams BL Irvin BJ Sutor SL Chini CC Yacyshyn E Bubeck Wardenburg J. Phosphorylation of Tyr319 in ZAP-70 Is Required for T-Cell Antigen Receptor-Dependent Phospholipase C-Gamma1 and Ras Activation. EMBO J (1999) 18(7):1832–44. doi: 10.1093/emboj/18.7.1832 PMC1171269 10202147

[33] Fisher WG Yang PC Medikonduri RK Jafri MS. NFAT and NFkappaB Activation in T Lymphocytes: A Model of Differential Activation of Gene Expression. Ann BioMed Eng (2006) 34(11):1712–28. doi: 10.1007/s10439-006-9179-4 PMC1764593 17031595

[34] Jamieson C McCaffrey PG Rao A Sen R. Physiologic Activation of T Cells *via* the T Cell Receptor Induces NF-Kappa B. J Immunol (Baltimore Md: 1950) (1991) 147(2):416–20. 1830061

[35] Verweij CL Geerts M Aarden LA. Activation of Interleukin-2 Gene-Transcription *Via* the T-Cell Surface-Molecule Cd28 Is Mediated Through an Nf-Kb-Like Response Element. J Biol Chem (1991) 266(22):14179–82. doi: 10.1016/S0021-9258(18)98663-1 1650350

[36] Shaw JP Utz PJ Durand DB Toole JJ Emmel EA Crabtree GR. Identification of a Putative Regulator of Early T-Cell Activation Genes. Science (1988) 241(4862):202–5. doi: 10.1126/science.3260404 3260404

[37] Rincón M Flavell RA. AP-1 Transcriptional Activity Requires Both T-Cell Receptor-Mediated and Co-Stimulatory Signals in Primary T Lymphocytes. EMBO J (1994) 13(18):4370–81. doi: 10.1002/j.1460-2075.1994.tb06757.x PMC395364 7925281

[38] Ross SH Cantrell DA. Signaling and Function of Interleukin-2 in T Lymphocytes. Annu Rev Immunol (2018) 36:411–33. doi: 10.1146/annurev-immunol-042617-053352 PMC6472684 29677473

[39] Esensten JH Helou YA Chopra G Weiss A Bluestone JA. CD28 Costimulation: From Mechanism to Therapy. Immunity (2016) 44(5):973–88. doi: 10.1016/j.immuni.2016.04.020 PMC4932896 27192564

[40] Frauwirth KA Riley JL Harris MH Parry RV Rathmell JC Plas DR. The CD28 Signaling Pathway Regulates Glucose Metabolism. Immunity (2002) 16(6):769–77. doi: 10.1016/S1074-7613(02)00323-0 12121659

[41] Garçon F Patton DT Emery JL Hirsch E Rottapel R Sasaki T. CD28 Provides T-Cell Costimulation and Enhances PI3K Activity at the Immune Synapse Independently of Its Capacity to Interact With the P85/P110 Heterodimer. Blood (2008) 111(3):1464–71. doi: 10.1182/blood-2007-08-108050 18006698

[42] Wülfing C Tunbridge HM Wraith DC. New Inhibitory Signaling by CTLA-4. Nat Immunol (2014) 15(5):408–9. doi: 10.1038/ni.2870 24747703

[43] Rudd CE Taylor A Schneider H. CD28 and CTLA-4 Coreceptor Expression and Signal Transduction. Immunol Rev (2009) 229(1):12–26. doi: 10.1111/j.1600-065X.2009.00770.x 19426212 PMC4186963

[44] Mukherjee S Maiti PK Nandi D. Role of CD80, CD86, and CTLA4 on Mouse CD4(+) T Lymphocytes in Enhancing Cell-Cycle Progression and Survival After Activation With PMA and Ionomycin. J Leukoc Biol (2002) 72(5):921–31. 12429713

[45] Wherry EJ Kurachi M. Molecular and Cellular Insights Into T Cell Exhaustion. Nat Rev Immunol (2015) 15(8):486–99. doi: 10.1038/nri3862 PMC4889009 26205583

[46] Guntermann C Alexander DR. CTLA-4 Suppresses Proximal TCR Signaling in Resting Human CD4(+) T Cells by Inhibiting ZAP-70 Tyr(319) Phosphorylation: A Potential Role for Tyrosine Phosphatases. J Immunol (Baltimore Md: 1950) (2002) 168(9):4420–9. doi: 10.4049/jimmunol.168.9.4420 11970985

[47] Brunner-Weinzierl MC Rudd CE. CTLA-4 and PD-1 Control of T-Cell Motility and Migration: Implications for Tumor Immunotherapy. Front Immunol (2018) 9:2737. doi: 10.3389/fimmu.2018.02737 30542345 PMC6277866

[48] Blank CU Haining WN Held W Hogan PG Kallies A Lugli E. Defining ‘T Cell Exhaustion’. Nat Rev Immunol (2019) 19(11):665–74. doi: 10.1038/s41577-019-0221-9 PMC7286441 31570879

[49] Frauwirth KA Thompson CB. Regulation of T Lymphocyte Metabolism. J Immunol (Baltimore Md: 1950) (2004) 172(8):4661–5. doi: 10.4049/jimmunol.172.8.4661 15067038

[50] Menk AV Scharping NE Moreci RS Zeng X Guy C Salvatore S. Early TCR Signaling Induces Rapid Aerobic Glycolysis Enabling Distinct Acute T Cell Effector Functions. Cell Rep (2018) 22(6):1509–21. doi: 10.1016/j.celrep.2018.01.040 PMC5973810 29425506

[51] Wolf T Jin W Zoppi G Vogel IA Akhmedov M Bleck CKE. Dynamics in Protein Translation Sustaining T Cell Preparedness. Nat Immunol (2020) 21(8):927–37. doi: 10.1038/s41590-020-0714-5 PMC7610365 32632289

[52] Almeida L Lochner M Berod L Sparwasser T. Metabolic Pathways in T Cell Activation and Lineage Differentiation. Semin Immunol (2016) 28(5):514–24. doi: 10.1016/j.smim.2016.10.009 27825556

[53] Boubali S Liopeta K Virgilio L Thyphronitis G Mavrothalassitis G Dimitracopoulos G. Calcium/calmodulin-Dependent Protein Kinase II Regulates IL-10 Production by Human T Lymphocytes: A Distinct Target in the Calcium Dependent Pathway. Mol Immunol (2012) 52(2):51–60. doi: 10.1016/j.molimm.2012.04.008 22578382

[54] Racioppi L Means AR. Calcium/calmodulin-Dependent Protein Kinase Kinase 2: Roles in Signaling and Pathophysiology. J Biol Chem (2012) 287(38):31658–65. doi: 10.1074/jbc.R112.356485 PMC3442500 22778263

[55] Tamás P Hawley SA Clarke RG Mustard KJ Green K Hardie DG. Regulation of the Energy Sensor AMP-Activated Protein Kinase by Antigen Receptor and Ca2+ in T Lymphocytes. J Exp Med (2006) 203(7):1665–70. doi: 10.1084/jem.20052469 PMC2118355 16818670

[56] Hardie DG Sakamoto K. AMPK: A Key Sensor of Fuel and Energy Status in Skeletal Muscle. Physiology (Bethesda) (2006) 21:48–60. doi: 10.1152/physiol.00044.2005 16443822

[57] Mondino A Mueller DL. mTOR at the Crossroads of T Cell Proliferation and Tolerance. Semin Immunol (2007) 19(3):162–72. doi: 10.1016/j.smim.2007.02.008 PMC1995654 17383196

[58] Pollizzi KN Patel CH Sun IH Oh MH Waickman AT Wen J. mTORC1 and mTORC2 Selectively Regulate CD8⁺ T Cell Differentiation. J Clin Invest (2015) 125(5):2090–108. doi: 10.1172/JCI77746 PMC4463194 25893604

[59] Liu C Chapman NM Karmaus PW Zeng H Chi H. mTOR and Metabolic Regulation of Conventional and Regulatory T Cells. J Leukoc Biol (2015) 97(5):837–47. doi: 10.1189/jlb.2RI0814-408R PMC4398256 25714803

[60] Pollizzi KN Patel CH Sun IH Oh MH Waickman AT Wen J. mTORC1 and mTORC2 Selectively Regulate CD8(+) T Cell Differentiation. J Clin Invest (2015) 125(5):2090–108. doi: 10.1172/JCI77746 PMC4463194 25893604

[61] Zhang L Tschumi BO Lopez-Mejia IC Oberle SG Meyer M Samson G. Mammalian Target of Rapamycin Complex 2 Controls CD8 T Cell Memory Differentiation in a Foxo1-Dependent Manner. Cell Rep (2016) 14(5):1206–17. doi: 10.1016/j.celrep.2015.12.095 26804903

[62] Gottlob K Majewski N Kennedy S Kandel E Robey RB Hay N. Inhibition of Early Apoptotic Events by Akt/PKB Is Dependent on the First Committed Step of Glycolysis and Mitochondrial Hexokinase. Genes Dev (2001) 15(11):1406–18. doi: 10.1101/gad.889901 PMC312709 11390360

[63] Kohn AD Summers SA Birnbaum MJ Roth RA. Expression of a Constitutively Active Akt Ser/Thr Kinase in 3T3-L1 Adipocytes Stimulates Glucose Uptake and Glucose Transporter 4 Translocation. J Biol Chem (1996) 271(49):31372–8. doi: 10.1074/jbc.271.49.31372 8940145

[64] Houddane A Bultot L Novellasdemunt L Johanns M Gueuning M-A Vertommen D. Role of Akt/PKB and PFKFB Isoenzymes in the Control of Glycolysis, Cell Proliferation and Protein Synthesis in Mitogen-Stimulated Thymocytes. Cell Signal (2017) 34:23–37. doi: 10.1016/j.cellsig.2017.02.019 28235572

[65] Wu S Yin X Fang X Zheng J Li L Liu X. C-MYC Responds to Glucose Deprivation in a Cell-Type-Dependent Manner. Cell Death Discov (2015) 1:15057–. doi: 10.1038/cddiscovery.2015.57 PMC4979460 27551483

[66] Peng M Yin N Chhangawala S Xu K Leslie CS Li MO. Aerobic Glycolysis Promotes T Helper 1 Cell Differentiation Through an Epigenetic Mechanism. Science (2016) 354(6311):481–4. doi: 10.1126/science.aaf6284 PMC5539971 27708054

[67] Vander Heiden MG Cantley LC Thompson CB. Understanding the Warburg Effect: The Metabolic Requirements of Cell Proliferation. Science (2009) 324(5930):1029–33. doi: 10.1126/science.1160809 PMC2849637 19460998

[68] Abdel-Haleem AM Lewis NE Jamshidi N Mineta K Gao X Gojobori T. The Emerging Facets of Non-Cancerous Warburg Effect. Front Endocrinol (Lausanne) (2017) 8:279. doi: 10.3389/fendo.2017.00279 29109698 PMC5660072

[69] Angiari S Runtsch MC Sutton CE Palsson-McDermott EM Kelly B Rana N. Pharmacological Activation of Pyruvate Kinase M2 Inhibits CD4(+) T Cell Pathogenicity and Suppresses Autoimmunity. Cell Metab (2020) 31(2):391–405.e8. doi: 10.1016/j.cmet.2019.10.015 31761564 PMC7001035

[70] Cantó C Menzies KJ Auwerx J. NAD(+) Metabolism and the Control of Energy Homeostasis: A Balancing Act Between Mitochondria and the Nucleus. Cell Metab (2015) 22(1):31–53. doi: 10.1016/j.cmet.2015.05.023 26118927 PMC4487780

[71] Gerriets VA Kishton RJ Nichols AG Macintyre AN Inoue M Ilkayeva O. Metabolic Programming and PDHK1 Control CD4+ T Cell Subsets and Inflammation. J Clin Invest (2015) 125(1):194–207. doi: 10.1172/JCI76012 25437876 PMC4382238

[72] Finlay DK. Starved Human T Lymphocytes Keep Fighting. Eur J Immunol (2015) 45(9):2480–3. doi: 10.1002/eji.201545885 26256443

[73] Johnson MO Wolf MM Madden MZ Andrejeva G Sugiura A Contreras DC. Distinct Regulation of Th17 and Th1 Cell Differentiation by Glutaminase-Dependent Metabolism. Cell (2018) 175(7):1780–95.e19. doi: 10.1016/j.cell.2018.10.001 30392958 PMC6361668

[74] Nakaya M Xiao YC Zhou XF Chang JH Chang M Cheng XH. Inflammatory T Cell Responses Rely on Amino Acid Transporter ASCT2 Facilitation of Glutamine Uptake and mTORC1 Kinase Activation. Immunity (2014) 40(5):692–705. doi: 10.1016/j.immuni.2014.04.007 24792914 PMC4074507

[75] Wang R Dillon CP Shi LZ Milasta S Carter R Finkelstein D. The Transcription Factor Myc Controls Metabolic Reprogramming Upon T Lymphocyte Activation. Immunity (2011) 35(6):871–82. doi: 10.1016/j.immuni.2011.09.021 PMC3248798 22195744

[76] Choi YH Jin N Kelly F Sakthivel SK Yu T. Elevation of Alanine Aminotransferase Activity Occurs After Activation of the Cell-Death Signaling Initiated by Pattern-Recognition Receptors But Before Activation of Cytolytic Effectors in NK or CD8+ T Cells in the Liver During Acute HCV Infection. PloS One (2016) 11(10):e0165533. doi: 10.1371/journal.pone.0165533 27788241 PMC5082795

[77] Bietz A Zhu HY Xue MM Xu CQ. Cholesterol Metabolism in T Cells. Front Immunol (2017) 8:1664. doi: 10.3389/fimmu.2017.01664 29230226 PMC5711771

[78] Gruenbacher G Thurnher M. Mevalonate Metabolism in Immuno-Oncology. Front Immunol (2017) 8:1714. doi: 10.3389/fimmu.2017.01714 29250078 PMC5717006

[79] Patsoukis N Bardhan K Weaver J Herbel C Seth P Li L. The Role of Metabolic Reprogramming in T Cell Fate and Function. Curr Trends Immunol (2016) 17:1–12. 28356677 PMC5367635

[80] DeBerardinis RJ Mancuso A Daikhin E Nissim I Yudkoff M Wehrli S. Beyond Aerobic Glycolysis: Transformed Cells can Engage in Glutamine Metabolism That Exceeds the Requirement for Protein and Nucleotide Synthesis. Proc Natl Acad Sci USA (2007) 104(49):19345–50. doi: 10.1073/pnas.0709747104 PMC2148292 18032601

[81] Gnanaprakasam JNR Wu RH Wang RN. Metabolic Reprogramming in Modulating T Cell Reactive Oxygen Species Generation and Antioxidant Capacity. Front Immunol (2018) 9:1–19. doi: 10.3389/fimmu.2018.01075 PMC5964129 29868027

[82] Altman BJ Stine ZE Dang CV. From Krebs to Clinic: Glutamine Metabolism to Cancer Therapy. Nat Rev Cancer (2016) 16(10):619–34. doi: 10.1038/nrc.2016.71 PMC5484415 27492215

[83] Hesterberg RS Cleveland JL Epling-Burnette PK. Role of Polyamines in Immune Cell Functions. Med Sci (Basel) (2018) 6(1). doi: 10.3390/medsci6010022 PMC5872179 29517999

[84] Dimeloe S Burgener AV Grählert J Hess C. T-Cell Metabolism Governing Activation, Proliferation and Differentiation; a Modular View. Immunology (2017) 150(1):35–44. doi: 10.1111/imm.12655 27479920 PMC5341500

[85] Kaadige MR Looper RE Kamalanaadhan S Ayer DE. Glutamine-Dependent Anapleurosis Dictates Glucose Uptake and Cell Growth by Regulating MondoA Transcriptional Activity. Proc Natl Acad Sci USA (2009) 106(35):14878–83. doi: 10.1073/pnas.0901221106 PMC2736411 19706488

[86] Reid MA Lowman XH Pan M Tran TQ Warmoes MO Ishak Gabra MB. Ikkβ Promotes Metabolic Adaptation to Glutamine Deprivation *via* Phosphorylation and Inhibition of PFKFB3. Genes Dev (2016) 30(16):1837–51. doi: 10.1101/gad.287235.116 PMC5024682 27585591

[87] Sener Z Cederkvist FH Volchenkov R Holen HL Skålhegg BS. T Helper Cell Activation and Expansion Is Sensitive to Glutaminase Inhibition Under Both Hypoxic and Normoxic Conditions. PloS One (2016) 11(7):e0160291. doi: 10.1371/journal.pone.0160291 27467144 PMC4965213

[88] Wu J Li G Li L Li D Dong Z Jiang P. Asparagine Enhances LCK Signalling to Potentiate CD8+ T-Cell Activation and Anti-Tumour Responses. Nat Cell Biol (2021) 23(1):75–86. doi: 10.1038/s41556-020-00615-4 33420490

[89] Pavlova NN Hui S Ghergurovich JM Fan J Intlekofer AM White RM. As Extracellular Glutamine Levels Decline, Asparagine Becomes an Essential Amino Acid. Cell Metab (2018) 27(2):428–38.e5. doi: 10.1016/j.cmet.2017.12.006 29337136 PMC5803449

[90] Huang H Vandekeere S Kalucka J Bierhansl L Zecchin A Brüning U. Role of Glutamine and Interlinked Asparagine Metabolism in Vessel Formation. EMBO J (2017) 36(16):2334–52. doi: 10.15252/embj.201695518 PMC5556263 28659375

[91] Luo M Brooks M Wicha MS. Asparagine and Glutamine: Co-Conspirators Fueling Metastasis. Cell Metab (2018) 27(5):947–9. doi: 10.1016/j.cmet.2018.04.012 29719230

[92] Geiger R Rieckmann JC Wolf T Basso C Feng Y Fuhrer T. L-Arginine Modulates T Cell Metabolism and Enhances Survival and Anti-Tumor Activity. Cell (2016) 167(3):829–42.e13. doi: 10.1016/j.cell.2016.09.031 27745970 PMC5075284

[93] Levring TB Hansen AK Nielsen BL Kongsbak M von Essen MR Woetmann A. Activated Human CD4+ T Cells Express Transporters for Both Cysteine and Cystine. Sci Rep (2012) 2(1):266. doi: 10.1038/srep00266 22355778 PMC3278673

[94] Ananieva EA Powell JD Hutson SM. Leucine Metabolism in T Cell Activation: mTOR Signaling and Beyond. Adv Nutr (2016) 7(4):798s–805s. doi: 10.3945/an.115.011221 27422517 PMC4942864

[95] Wolfson RL Chantranupong L Saxton RA Shen K Scaria SM Cantor JR. Sestrin2 Is a Leucine Sensor for the mTORC1 Pathway. Science (2016) 351(6268):43–8. doi: 10.1126/science.aab2674 PMC4698017 26449471

[96] Sinclair LV Rolf J Emslie E Shi YB Taylor PM Cantrell DA. Control of Amino-Acid Transport by Antigen Receptors Coordinates the Metabolic Reprogramming Essential for T Cell Differentiation. Nat Immunol (2013) 14(5):500–8. doi: 10.1038/ni.2556 PMC3672957 23525088

[97] Ma EH Bantug G Griss T Condotta S Johnson RM Samborska B. Serine Is an Essential Metabolite for Effector T Cell Expansion. Cell Metab (2017) 25(2):482. doi: 10.1016/j.cmet.2016.12.011 28178570

[98] McNamee EN Korns Johnson D Homann D Clambey ET. Hypoxia and Hypoxia-Inducible Factors as Regulators of T Cell Development, Differentiation, and Function. Immunol Res (2013) 55(1-3):58–70. doi: 10.1007/s12026-012-8349-8 22961658 PMC3919451

[99] Braun RD Lanzen JL Snyder SA Dewhirst MW. Comparison of Tumor and Normal Tissue Oxygen Tension Measurements Using OxyLite or Microelectrodes in Rodents. Am J Physiol Heart Circ Physiol (2001) 280(6):H2533–44. doi: 10.1152/ajpheart.2001.280.6.H2533 11356608

[100] Hale LP Braun RD Gwinn WM Greer PK Dewhirst MW. Hypoxia in the Thymus: Role of Oxygen Tension in Thymocyte Survival. Am J Physiol Heart Circ Physiol (2002) 282(4):H1467–H77. doi: 10.1152/ajpheart.00682.2001 11893584

[101] Biniecka M Canavan M McGarry T Gao W McCormick J Cregan S. Dysregulated Bioenergetics: A Key Regulator of Joint Inflammation. Ann Rheum Dis (2016) 75(12):2192–200. doi: 10.1136/annrheumdis-2015-208476 PMC5136702 27013493

[102] Tao JH Barbi J Pan F. Hypoxia-Inducible Factors in T Lymphocyte Differentiation and Function. A Review in the Theme: Cellular Responses to Hypoxia. Am J Physiol Cell Physiol (2015) 309(9):C580–9. doi: 10.1152/ajpcell.00204.2015 PMC4628938 26354751

[103] Serganova I Cohen IJ Vemuri K Shindo M Maeda M Mane M. LDH-A Regulates the Tumor Microenvironment *via* HIF-Signaling and Modulates the Immune Response. PloS One (2018) 13(9):1–22. doi: 10.1371/journal.pone.0203965 PMC6153000 30248111

[104] Kim JW Tchernyshyov I Semenza GL Dang CV. HIF-1-Mediated Expression of Pyruvate Dehydrogenase Kinase: A Metabolic Switch Required for Cellular Adaptation to Hypoxia. Cell Metab (2006) 3(3):177–85. doi: 10.1016/j.cmet.2006.02.002 16517405

[105] Tyrakis PA Palazon A Macias D Lee KL Phan AT Veliça P. S-2-Hydroxyglutarate Regulates CD8+ T-Lymphocyte Fate. Nature (2016) 540(7632):236–41. doi: 10.1038/nature20165 PMC5149074 27798602

[106] Böttcher M Renner K Berger R Mentz K Thomas S Cardenas-Conejo ZE. D-2-Hydroxyglutarate Interferes With HIF-1α Stability Skewing T-Cell Metabolism Towards Oxidative Phosphorylation and Impairing Th17 Polarization. OncoImmunology (2018) 7(7):e1445454. doi: 10.1080/2162402X.2018.1445454 29900057 PMC5993507

[107] Geltink RIK Kyle RL Pearce EL. Unraveling the Complex Interplay Between T Cell Metabolism and Function. Annu Rev Immunol (2018) 36:461–88. doi: 10.1146/annurev-immunol-042617-053019 PMC6323527 29677474

[108] Shen Y Wen Z Li Y Matteson EL Hong J Goronzy JJ. Metabolic Control of the Scaffold Protein TKS5 in Tissue-Invasive, Proinflammatory T Cells. Nat Immunol (2017) 18(9):1025–34. doi: 10.1038/ni.3808 PMC5568495 28737753

[109] Kono M Yoshida N Maeda K Tsokos GC. Transcriptional Factor ICER Promotes Glutaminolysis and the Generation of Th17 Cells. Proc Natl Acad Sci (2018) 115(10):2478–83. doi: 10.1073/pnas.1714717115 PMC5877961 29463741

[110] Chang C-H Curtis Jonathan D Maggi Leonard B Faubert B Villarino Alejandro V O’Sullivan D. Posttranscriptional Control of T Cell Effector Function by Aerobic Glycolysis. Cell (2013) 153(6):1239–51. doi: 10.1016/j.cell.2013.05.016 PMC3804311 23746840

[111] Guppy M Greiner E Brand K. The Role of the Crabtree Effect and an Endogenous Fuel in the Energy Metabolism of Resting and Proliferating Thymocytes. Eur J Biochem (1993) 212(1):95–9. doi: 10.1111/j.1432-1033.1993.tb17637.x 8444168

[112] Vrisekoop N den Braber I de Boer AB Ruiter AFC Ackermans MT van der Crabben SN. Sparse Production But Preferential Incorporation of Recently Produced Naive T Cells in the Human Peripheral Pool. Proc Natl Acad Sci U S A (2008) 105(16):6115–20. doi: 10.1073/pnas.0709713105 PMC2329696 18420820

[113] Michalek RD Gerriets VA Jacobs SR Macintyre AN MacIver NJ Mason EF. Cutting Edge: Distinct Glycolytic and Lipid Oxidative Metabolic Programs Are Essential for Effector and Regulatory CD4+ T Cell Subsets. J Immunol (2011) 186(6):3299–303. doi: 10.4049/jimmunol.1003613 PMC3198034 21317389

[114] Zygmunt BM Węgrzyn A Gajska W Yevsa T Chodaczek G Guzmán CA. Mannose Metabolism Is Essential for Th1 Cell Differentiation and IFN-γ Production. J Immunol (2018) 201(5):1400–11. doi: 10.4049/jimmunol.1700042 30030325

[115] Lee K Gudapati P Dragovic S Spencer C Joyce S Killeen N. Mammalian Target of Rapamycin Protein Complex 2 Regulates Differentiation of Th1 and Th2 Cell Subsets *via* Distinct Signaling Pathways. Immunity (2010) 32(6):743–53. doi: 10.1016/j.immuni.2010.06.002 PMC2911434 20620941

[116] Hernandez-Quiles M Broekema MF Kalkhoven E. PPARgamma in Metabolism, Immunity, and Cancer: Unified and Diverse Mechanisms of Action. Front Endocrinol (2021) 12:624112. doi: 10.3389/fendo.2021.624112 PMC7953066 33716977

[117] Dang EV Barbi J Yang H-Y Jinasena D Yu H Zheng Y. Control of TH17/Treg Balance by Hypoxia-Inducible Factor 1. Cell (2011) 146(5):772–84. doi: 10.1016/j.cell.2011.07.033 PMC3387678 21871655

[118] Berod L Friedrich C Nandan A Freitag J Hagemann S Harmrolfs K. *De Novo* Fatty Acid Synthesis Controls the Fate Between Regulatory T and T Helper 17 Cells. Nat Med (2014) 20(11):1327–33. doi: 10.1038/nm.3704 25282359

[119] Wang Y Bi Y Chen X Li C Li Y Zhang Z. Histone Deacetylase SIRT1 Negatively Regulates the Differentiation of Interleukin-9-Producing CD4+ T Cells. Immunity (2016) 44(6):1337–49. doi: 10.1016/j.immuni.2016.05.009 27317260

[120] Cluxton D Petrasca A Moran B Fletcher JM. Differential Regulation of Human Treg and Th17 Cells by Fatty Acid Synthesis and Glycolysis. Front Immunol (2019) 10:115. doi: 10.3389/fimmu.2019.00115 30778354 PMC6369198

[121] Shi LZ Wang R Huang G Vogel P Neale G Green DR. HIF1alpha-Dependent Glycolytic Pathway Orchestrates a Metabolic Checkpoint for the Differentiation of TH17 and Treg Cells. J Exp Med (2011) 208(7):1367–76. doi: 10.1084/jem.20110278 PMC3135370 21708926

[122] Gerriets VA Kishton RJ Johnson MO Cohen S Siska PJ Nichols AG. Foxp3 and Toll-Like Receptor Signaling Balance T(reg) Cell Anabolic Metabolism for Suppression. Nat Immunol (2016) 17(12):1459–66. doi: 10.1038/ni.3577 PMC5215903 27695003

[123] Watson MJ Vignali PDA Mullett SJ Overacre-Delgoffe AE Peralta RM Grebinoski S. Metabolic Support of Tumour-Infiltrating Regulatory T Cells by Lactic Acid. Nature (2021) 591(7851):645–51. doi: 10.1038/s41586-020-03045-2 PMC7990682 33589820

[124] Kempkes RWM Joosten I Koenen HJPM He X. Metabolic Pathways Involved in Regulatory T Cell Functionality. Front Immunol (2019) 10:2839. doi: 10.3389/fimmu.2019.02839 31849995 PMC6902900

[125] O’Sullivan D. The Metabolic Spectrum of Memory T Cells. Immunol Cell Biol (2019) 97(7):636–46. doi: 10.1111/imcb.12274 31127964

[126] Wu H Estrella V Beatty M Abrahams D El-Kenawi A Russell S. T-Cells Produce Acidic Niches in Lymph Nodes to Suppress Their Own Effector Functions. Nat Commun (2020) 11(1):4113. doi: 10.1038/s41467-020-17756-7 32807791 PMC7431837

[127] Allison KE Coomber BL Bridle BW. Metabolic Reprogramming in the Tumour Microenvironment: A Hallmark Shared by Cancer Cells and T Lymphocytes. Immunology (2017) 152(2):175–84. doi: 10.1111/imm.12777 PMC5588769 28621843

[128] Binnewies M Roberts EW Kersten K Chan V Fearon DF Merad M. Understanding the Tumor Immune Microenvironment (TIME) for Effective Therapy. Nat Med (2018) 24(5):541–50. doi: 10.1038/s41591-018-0014-x PMC5998822 29686425

[129] Certo M Tsai C-H Pucino V Ho P-C Mauro C. Lactate Modulation of Immune Responses in Inflammatory Versus Tumour Microenvironments. Nat Rev Immunol (2021) 21(3):151–61. doi: 10.1038/s41577-020-0406-2 32839570

[130] de la Cruz-López KG Castro-Muñoz LJ Reyes-Hernández DO García-Carrancá A Manzo-Merino J. Lactate in the Regulation of Tumor Microenvironment and Therapeutic Approaches. Front Oncol (2019) 9:1143. doi: 10.3389/fonc.2019.01143 31737570 PMC6839026

[131] Pucino V Certo M Bulusu V Cucchi D Goldmann K Pontarini E. Lactate Buildup at the Site of Chronic Inflammation Promotes Disease by Inducing CD4+ T Cell Metabolic Rewiring. Cell Metab (2019) 30(6):1055–74.e8. doi: 10.1016/j.cmet.2019.10.004 31708446 PMC6899510

[132] Evans SS Repasky EA Fisher DT. Fever and the Thermal Regulation of Immunity: The Immune System Feels the Heat. Nat Rev Immunol (2015) 15(6):335–49. doi: 10.1038/nri3843 PMC4786079 25976513

[133] O’Sullivan D Stanczak MA Villa M Uhl FM Corrado M Klein Geltink RI. Fever Supports CD8+ Effector T Cell Responses by Promoting Mitochondrial Translation. Proc Natl Acad Sci (2021) 118(25):e2023752118. doi: 10.1073/pnas.2023752118 34161266 PMC8237659

[134] Kahan SM Wherry EJ Zajac AJ. T Cell Exhaustion During Persistent Viral Infections. Virology (2015) 479-480:180–93. doi: 10.1016/j.virol.2014.12.033 PMC4424083 25620767

[135] Franco F Jaccard A Romero P Yu Y-R Ho P-C. Metabolic and Epigenetic Regulation of T-Cell Exhaustion. Nat Metab (2020) 2(10):1001–12. doi: 10.1038/s42255-020-00280-9 32958939

[136] Kolan SS Li G Wik JA Malachin G Guo S Kolan P. Cellular Metabolism Dictates T Cell Effector Function in Health and Disease. Scand J Immunol (2020) 92(5):e12956. doi: 10.1111/sji.12956 32767795

[137] Reina-Campos M Scharping NE Goldrath AW. CD8+ T Cell Metabolism in Infection and Cancer. Nat Rev Immunol (2021) 21(11):718–38. doi: 10.1038/s41577-021-00537-8 PMC8806153 33981085

[138] Guglani L Khader SA. Th17 Cytokines in Mucosal Immunity and Inflammation. Curr Opin HIV AIDS (2010) 5(2):120–7. doi: 10.1097/COH.0b013e328335c2f6 PMC2892849 20543588

[139] Zhang Q Xiang R Huo S Zhou Y Jiang S Wang Q. Molecular Mechanism of Interaction Between SARS-CoV-2 and Host Cells and Interventional Therapy. Signal Transduct Target Ther (2021) 6(1):233. doi: 10.1038/s41392-021-00653-w 34117216 PMC8193598

[140] Lokhande AS Devarajan PV. A Review on Possible Mechanistic Insights of Nitazoxanide for Repurposing in COVID-19. Eur J Pharmacol (2021) 891:173748. doi: 10.1016/j.ejphar.2020.173748 33227285 PMC7678434

[141] Mayi BS Leibowitz JA Woods AT Ammon KA Liu AE Raja A. The Role of Neuropilin-1 in COVID-19. PloS Pathog (2021) 17(1):e1009153. doi: 10.1371/journal.ppat.1009153 33395426 PMC7781380

[142] Wang S Qiu Z Hou Y Deng X Xu W Zheng T. AXL Is a Candidate Receptor for SARS-CoV-2 That Promotes Infection of Pulmonary and Bronchial Epithelial Cells. Cell Res (2021) 31(2):126–40. doi: 10.1038/s41422-020-00460-y PMC7791157 33420426

[143] Zhu C Wei Y Wei X. AXL Receptor Tyrosine Kinase as a Promising Anti-Cancer Approach: Functions, Molecular Mechanisms and Clinical Applications. Mol Cancer (2019) 18(1):153. doi: 10.1186/s12943-019-1090-3 31684958 PMC6827209

[144] Mullen PJ Garcia G Jr. Purkayastha A Matulionis N Schmid EW Momcilovic M. SARS-CoV-2 Infection Rewires Host Cell Metabolism and Is Potentially Susceptible to mTORC1 Inhibition. Nat Commun (2021) 12(1):1876–. doi: 10.1038/s41467-021-22166-4 PMC7994801 33767183

[145] Liu X Zhao J Wang H Wang W Su X Liao X. Metabolic Defects of Peripheral T Cells in COVID-19 Patients. J Immunol (2021) 206(12):2900–8. doi: 10.4049/jimmunol.2100068 34049969

[146] Jin C Luo X Qian S Zhang K Gao Y Zhou R. Positron Emission Tomography in the COVID-19 Pandemic Era. Eur J Nucl Med Mol Imaging (2021) 48(12):3903–17. doi: 10.1007/s00259-021-05347-7 PMC8134823 34013405

[147] Shi D Yan R Lv L Jiang H Lu Y Sheng J. The Serum Metabolome of COVID-19 Patients Is Distinctive and Predictive. Metabolism (2021) 118:154739. doi: 10.1016/j.metabol.2021.154739 33662365 PMC7920809

[148] Kumar V. How Could We Forget Immunometabolism in SARS-CoV2 Infection or COVID-19? Int Rev Immunol (2021) 40(1-2):72–107. doi: 10.1080/08830185.2020.1840567 33155525

[149] Ajaz S McPhail MJ Singh KK Mujib S Trovato FM Napoli S. Mitochondrial Metabolic Manipulation by SARS-CoV-2 in Peripheral Blood Mononuclear Cells of Patients With COVID-19. Am J Physiol Cell Physiol (2021) 320(1):C57–65. doi: 10.1152/ajpcell.00426.2020 PMC7816428 33151090

[150] Codo AC Davanzo GG Monteiro L de Souza GF Muraro SP Virgilio-da-Silva JV. Elevated Glucose Levels Favor SARS-CoV-2 Infection and Monocyte Response Through a HIF-1α/Glycolysis-Dependent Axis. Cell Metab (2020) 32(3):437–46.e5. doi: 10.2139/ssrn.3606770 32697943 PMC7367032

[151] Halder S Mehta AK. 2-Deoxy-D-Glucose: Is This the Final Cure for COVID-19: Or Yet Another Mirage? Eur Rev Med Pharmacol Sci (2021) 25(13):4448–50. doi: 10.26355/eurrev_202107_26234 34286486

[152] De Biasi S Meschiari M Gibellini L Bellinazzi C Borella R Fidanza L. Marked T Cell Activation, Senescence, Exhaustion and Skewing Towards TH17 in Patients With COVID-19 Pneumonia. Nat Commun (2020) 11(1):3434. doi: 10.21203/rs.3.rs-23957/v1 32632085 PMC7338513

[153] Siska PJ Decking S-M Babl N Matos C Bruss C Singer K. Metabolic Imbalance of T Cells in COVID-19 Is Hallmarked by Basigin and Mitigated by Dexamethasone. J Clin Invest (2021) 131(22):1–16. doi: 10.1172/JCI148225 PMC8592546 34779418

[154] Guo N Ye S Zhang K Yu X Cui H Yang X. A Critical Epitope in CD147 Facilitates Memory CD4+ T-Cell Hyper-Activation in Rheumatoid Arthritis. Cell Mol Immunol (2019) 16(6):568–79. doi: 10.1038/s41423-018-0012-4 PMC6804595 29563614

[155] Carfì A Bernabei R Landi F Gemelli Against C-P-ACSG. Persistent Symptoms in Patients After Acute COVID-19. JAMA (2020) 324(6):603–5. doi: 10.1001/jama.2020.12603 PMC7349096 32644129

[156] Havervall S Rosell A Phillipson M Mangsbo SM Nilsson P Hober S. Symptoms and Functional Impairment Assessed 8 Months After Mild COVID-19 Among Health Care Workers. JAMA (2021) 325(19):2015–6. doi: 10.1001/jama.2021.5612 PMC8027932 33825846

[157] Visvabharathy L Hanson B Orban Z Lim PH Palacio N Jain R. Neuro-COVID Long-Haulers Exhibit Broad Dysfunction in T Cell Memory Generation and Responses to Vaccination. medRxiv (2021) 2021.08.08.21261763. doi: 10.1101/2021.08.08.21261763

[158] Vehar S Boushra M Ntiamoah P Biehl M. Post-Acute Sequelae of SARS-CoV-2 Infection: Caring for the ‘Long-Haulers’. Cleve Clin J Med (2021) 88(5):267–72. doi: 10.3949/ccjm.88a.21010 33941600

[159] Thompson EA Cascino K Ordonez AA Zhou W Vaghasia A Hamacher-Brady A. Metabolic Programs Define Dysfunctional Immune Responses in Severe COVID-19 Patients. Cell Rep (2021) 34(11):1–19. doi: 10.1016/j.celrep.2021.108863 PMC7908880 33691089

[160] LaMere SA Thompson RC Meng X Komori HK Mark A Salomon DR. H3K27 Methylation Dynamics During CD4 T Cell Activation: Regulation of JAK/STAT and IL12RB2 Expression by JMJD3. J Immunol (Baltimore Md: 1950) (2017) 199(9):3158–75. doi: 10.4049/jimmunol.1700475 PMC5679303 28947543

[161] Cribbs AP Terlecki-Zaniewicz S Philpott M Baardman J Ahern D Lindow M. Histone H3K27me3 Demethylases Regulate Human Th17 Cell Development and Effector Functions by Impacting on Metabolism. Proc Natl Acad Sci (2020) 117(11):6056–66. doi: 10.1073/pnas.1919893117 PMC7084125 32123118

[162] Heming M Li X Räuber S Mausberg AK Börsch A-L Hartlehnert M. Neurological Manifestations of COVID-19 Feature T Cell Exhaustion and Dedifferentiated Monocytes in Cerebrospinal Fluid. Immunity (2021) 54(1):164–75.e6. doi: 10.1016/j.immuni.2020.12.011 33382973 PMC7831653

[163] Karki R Sharma BR Tuladhar S Williams EP Zalduondo L Samir P. Synergism of TNF-α and IFN-γ Triggers Inflammatory Cell Death, Tissue Damage, and Mortality in SARS-CoV-2 Infection and Cytokine Shock Syndromes. Cell (2021) 184(1):149–68.e17. doi: 10.1016/j.cell.2020.11.025 33278357 PMC7674074

[164] Walker B McMichael A. The T-Cell Response to HIV. Cold Spring Harb Perspect Med (2012) 2(11):a007054. doi: 10.1101/cshperspect.a007054 23002014 PMC3543107

[165] Vidya Vijayan KK Karthigeyan KP Tripathi SP Hanna LE. Pathophysiology of CD4+ T-Cell Depletion in HIV-1 and HIV-2 Infections. Front Immunol (2017) 8:580. doi: 10.3389/fimmu.2017.00580 28588579 PMC5440548

[166] Porichis F Kaufmann DE. Role of PD-1 in HIV Pathogenesis and as Target for Therapy. Curr HIV/AIDS Rep (2012) 9(1):81–90. doi: 10.1007/s11904-011-0106-4 22198819 PMC3731769

[167] Fenwick C Joo V Jacquier P Noto A Banga R Perreau M. T-Cell Exhaustion in HIV Infection. Immunol Rev (2019) 292(1):149–63. doi: 10.1111/imr.12823 PMC7003858 31883174

[168] Choi W-T Yang Y Xu Y An J. Targeting Chemokine Receptor CXCR4 for Treatment of HIV-1 Infection, Tumor Progression, and Metastasis. Curr Top Med Chem (2014) 14(13):1574–89. doi: 10.2174/1568026614666140827143541 PMC4372248 25159167

[169] Loisel-Meyer S Swainson L Craveiro M Oburoglu L Mongellaz C Costa C. Glut1-Mediated Glucose Transport Regulates HIV Infection. Proc Natl Acad Sci USA (2012) 109(7):2549–54. doi: 10.1073/pnas.1121427109 PMC3289356 22308487

[170] Palmer CS Ostrowski M Gouillou M Tsai L Yu D Zhou J. Increased Glucose Metabolic Activity Is Associated With CD4+ T-Cell Activation and Depletion During Chronic HIV Infection. AIDS (2014) 28(3):297–309. doi: 10.1097/QAD.0000000000000128 24335483 PMC4293200

[171] Guo H Wang Q Ghneim K Wang L Rampanelli E Holley-Guthrie E. Multi-Omics Analyses Reveal That HIV-1 Alters CD4+ T Cell Immunometabolism to Fuel Virus Replication. Nat Immunol (2021) 22(4):423–33. doi: 10.1038/s41590-021-00898-1 PMC8087183 33767427

[172] Hegedus A Kavanagh Williamson M Huthoff H. HIV-1 Pathogenicity and Virion Production Are Dependent on the Metabolic Phenotype of Activated CD4+ T Cells. Retrovirology (2014) 11:98. doi: 10.1186/s12977-014-0098-4 PMC4252996 25421745

[173] Angin M Volant S Passaes C Lecuroux C Monceaux V Dillies M-A. Metabolic Plasticity of HIV-Specific CD8+ T Cells Is Associated With Enhanced Antiviral Potential and Natural Control of HIV-1 Infection. Nat Metab (2019) 1(7):704–16. doi: 10.1038/s42255-019-0081-4 32694646

[174] Houben RM Dodd PJ. The Global Burden of Latent Tuberculosis Infection: A Re-Estimation Using Mathematical Modelling. PloS Med (2016) 13(10):e1002152. doi: 10.1371/journal.pmed.1002152 27780211 PMC5079585

[175] de Martino M Lodi L Galli L Chiappini E. Immune Response to Mycobacterium Tuberculosis: A Narrative Review. Front Pediatr (2019) 7:350. doi: 10.3389/fped.2019.00350 31508399 PMC6718705

[176] Geldmacher C Zumla A Hoelscher M. Interaction Between HIV and Mycobacterium Tuberculosis: HIV-1-Induced CD4 T-Cell Depletion and the Development of Active Tuberculosis. Curr Opin HIV AIDS (2012) 7(3):268–75. doi: 10.1097/COH.0b013e3283524e32 22495739

[177] Barber DL Mayer-Barber KD Feng CG Sharpe AH Sher A. CD4 T Cells Promote Rather Than Control Tuberculosis in the Absence of PD-1–Mediated Inhibition. J Immunol (2011) 186(3):1598–607. doi: 10.4049/jimmunol.1003304 PMC4059388 21172867

[178] Crowther RR Qualls JE. Metabolic Regulation of Immune Responses to Mycobacterium Tuberculosis: A Spotlight on L-Arginine and L-Tryptophan Metabolism. Front Immunol (2021) 11:628432. doi: 10.3389/fimmu.2020.628432 33633745 PMC7900187

[179] Schinzel AC Takeuchi O Huang Z Fisher JK Zhou Z Rubens J. Cyclophilin D Is a Component of Mitochondrial Permeability Transition and Mediates Neuronal Cell Death After Focal Cerebral Ischemia. Proc Natl Acad Sci USA (2005) 102(34):12005–10. doi: 10.1073/pnas.0505294102 PMC1189333 16103352

[180] Bernardi P Di Lisa F. The Mitochondrial Permeability Transition Pore: Molecular Nature and Role as a Target in Cardioprotection. J Mol Cell Cardiol (2015) 78:100–6. doi: 10.1016/j.yjmcc.2014.09.023 PMC4294587 25268651

[181] Tzelepis F Blagih J Khan N Gillard J Mendonca L Roy DG. Mitochondrial Cyclophilin D Regulates T Cell Metabolic Responses and Disease Tolerance to Tuberculosis. Sci Immunol (2018) 3(23):eaar4135. doi: 10.1126/sciimmunol.aar4135 29752301

[182] Russell SL Lamprecht DA Mandizvo T Jones TT Naidoo V Addicott KW. Compromised Metabolic Reprogramming Is an Early Indicator of CD8+ T Cell Dysfunction During Chronic Mycobacterium Tuberculosis Infection. Cell Rep (2019) 29(11):3564–79.e5. doi: 10.1016/j.celrep.2019.11.034 31825836 PMC6915325

[183] Yan Y Zhang G-X Gran B Fallarino F Yu S Li H. IDO Upregulates Regulatory T Cells *via* Tryptophan Catabolite and Suppresses Encephalitogenic T Cell Responses in Experimental Autoimmune Encephalomyelitis. J Immunol (Baltimore Md: 1950) (2010) 185(10):5953–61. doi: 10.4049/jimmunol.1001628 PMC2998795 20944000

[184] Adams KN Moguche AO Plumlee CR Urdahl KB. TGF-β-Mediated Inhibition of IFN-γ Production by Mycobacterium Tuberculosis-Specific T Cells in the Infected Lung. J Immunol (2016) 196(1 Supplement):65–5.

[185] Mehra S Alvarez X Didier PJ Doyle LA Blanchard JL Lackner AA. Granuloma Correlates of Protection Against Tuberculosis and Mechanisms of Immune Modulation by Mycobacterium Tuberculosis. J Infect Dis (2012) 207(7):1115–27. doi: 10.1093/infdis/jis778 PMC3633457 23255564

[186] Gautam US Foreman TW Bucsan AN Veatch AV Alvarez X Adekambi T. *In Vivo* Inhibition of Tryptophan Catabolism Reorganizes the Tuberculoma and Augments Immune-Mediated Control of Mycobacterium Tuberculosis. Proc Natl Acad Sci (2018) 115(1):E62–71. doi: 10.1073/pnas.1711373114 PMC5776797 29255022

[187] Mezrich JD Fechner JH Zhang X Johnson BP Burlingham WJ Bradfield CA. An Interaction Between Kynurenine and the Aryl Hydrocarbon Receptor Can Generate Regulatory T Cells. J Immunol (2010) 185(6):3190–8. doi: 10.4049/jimmunol.0903670 PMC2952546 20720200

[188] Renner K Bruss C Schnell A Koehl G Becker HM Fante M. Restricting Glycolysis Preserves T Cell Effector Functions and Augments Checkpoint Therapy. Cell Rep (2019) 29(1):135–50.e9. doi: 10.1016/j.celrep.2019.08.068 31577944

[189] Klysz D Tai X Robert PA Craveiro M Cretenet G Oburoglu L. Glutamine-Dependent α-Ketoglutarate Production Regulates the Balance Between T Helper 1 Cell and Regulatory T Cell Generation. Sci Signal (2015) 8(396):ra97–ra. doi: 10.1126/scisignal.aab2610 26420908

[190] Saeidi A Zandi K Cheok YY Saeidi H Wong WF Lee CYQ. T-Cell Exhaustion in Chronic Infections: Reversing the State of Exhaustion and Reinvigorating Optimal Protective Immune Responses. Front Immunol (2018) 9:2569. doi: 10.3389/fimmu.2018.02569 30473697 PMC6237934

